# Self‐Sustaining Sensing Systems: Integration Strategies and Signal–Power Matching in Energy‐Harvesting Electronics

**DOI:** 10.1002/advs.77712

**Published:** 2026-09-10

**Authors:** Taehyun Park, Ki‐Yoon Park, Yeonho Choi, Dong‐Myeong Shin, Hong‐Joon Yoon, Hocheon Yoo

**Affiliations:** ^1^ Department of Electronic Engineering Hanyang University Seoul Republic of Korea; ^2^ Department of Semiconductor Engineering Gachon University Seongnam Republic of Korea; ^3^ Department of Electronic Engineering Gachon University Seongnam Republic of Korea; ^4^ Department of Mechanical Engineering The University of Hong Kong Hong Kong SAR China; ^5^ Department of Artificial Intelligence Semiconductor Engineering Hanyang University Seoul Republic of Korea; ^6^ Hanyang Institute For Quantum Science and Quantum Technology Hanyang University Seoul Republic of Korea

**Keywords:** co‐functional devices, energy harvesting, event‐driven sensing, monolithic integration, multi‐modal sensing, self‐powered sensors, signal–power matching

## Abstract

Self‐sustaining sensing systems that simultaneously harvest ambient energy and perform real‐time signal acquisition are emerging as key technologies for wearable electronics, intelligent Internet‐of‐Things platforms, and autonomous edge systems. Despite rapid advances in individual energy harvesters and self‐powered sensors, a unified framework connecting device integration and operational strategy remains underdeveloped. In this review, we present systematic integration strategies and signal–power matching principles for energy harvester‐integrated sensing systems. First, energy harvester‐integrated platforms are categorized into three representative architectures: co‐packaged devices, monolithically integrated devices, and co‐functional devices, according to the physical and functional relationship between the harvester and sensing unit. Their structural characteristics, operational advantages, and representative implementations across photovoltaic, thermoelectric, triboelectric, and piezoelectric systems are comparatively discussed. Beyond structural integration, we further classify operational paradigms into always‐on sensing, event‐driven sensing, and multi‐modal sensing based on the temporal characteristics of sensed signals and harvested power profiles. This framework clarifies how harvested energy is distributed across sensing, storage, processing, and wireless communication pathways in practical autonomous systems. Key challenges and future opportunities toward scalable self‐sustaining intelligent sensing platforms are discussed.

## Graphical abstract

Energy‐harvesting‐integrated sensors couple ambient energy conversion with sensing, processing, and wireless communication. This review organizes recent progress through integration architectures and operating principles, revisiting co‐packaged, monolithic, and co‐functional designs together with always‐on, event‐driven, and multi‐modal sensing modes.

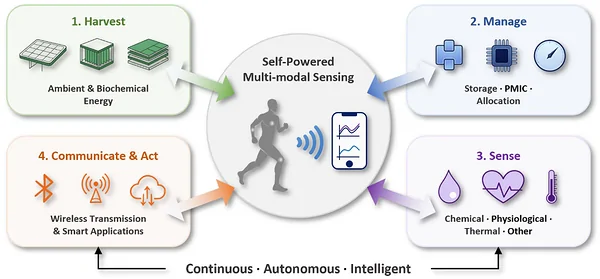

## 1 Introduction

Recent advances in electronic materials, device engineering, and miniaturized system design have rapidly expanded the capabilities of modern sensors beyond conventional signal detection [1, 2, 3]. A wide range of sensing technologies, including physiological [4, 5], chemical [6, 7], optical [8, 9], thermal [10], pressure [11, 12], and environmental sensors [13, 14], are now being actively developed for applications spanning wearable healthcare [15, 16], soft robotics [17, 18], industrial diagnostics [19], risk monitoring [20], and intelligent Internet‐of‐Things (IoT) platforms [21, 22]. Flexible and skin‐conformal sensors [23, 24] capable of continuously monitoring biological and environmental information in real time have attracted growing attention as next‐generation intelligent interfaces between humans, machines, and surrounding environments.

As sensing technologies continue to evolve, the role of sensors is also shifting from isolated stationary components toward distributed and continuously connected intelligent nodes. Modern sensing systems are increasingly expected to operate while moving with users, interacting with surrounding devices, and wirelessly transmitting acquired information in real time [25]. In this recent context, power sustainability has emerged as one of the most critical challenges limiting long‐term autonomous operation [26]. Conventional battery‐based approaches suffer from restricted lifetime [27, 28], periodic replacement requirements [29], increased device volume due to embedded batteries [30], and limited suitability [31] for flexible or wearable platforms. Modern environments are filled with overlooked streams of ambient energy that are constantly generated yet rarely utilized. Light, heat, vibration, motion, airflow, and even biochemical activity from the human body continuously surround us as potential energy resources [32, 33]. Using these ubiquitous energy sources directly as operational power for sensors offers an attractive route toward maintenance‐free and self‐sustaining sensing systems.

Can a sensor harvest energy and sense the environment at the same time? Driven by this question, energy harvesting (EH) technologies have recently evolved beyond their conventional role as standalone power sources toward multifunctional platforms capable of simultaneously supporting sensing, signal processing, and wireless communication [34, 35, 36]. For example, photovoltaic (PV) cells [37], thermoelectric generators (TEGs) [38], triboelectric nanogenerators (TENGs) [39], and piezoelectric nanogenerators (PENGs) [40] convert different forms of ambient energy into electricity through distinct physical mechanisms. More recently, these EH units have begun to be physically and functionally integrated with sensing components [41], which allows for compact and autonomous systems where energy generation and signal acquisition occur within a shared platform [42]. Recent developments have also extended self‐powered transduction toward chemically selective and molecular sensing, where analyte‐dependent interfacial interactions can be encoded directly into the generated electrical response [43, 44, 45]. Depending on how the harvesting and sensing units are organized, these systems can exhibit markedly different structural characteristics, operational mechanisms, and energy‐management pathways. Based on these perspectives, understanding how energy harvesters and sensors should be integrated and operated has emerged as a key challenge in the development of next‐generation self‐sustaining intelligent sensing systems.

Long‐term operation of EH‐integrated sensing systems depends on the coordinated design of energy harvesting, sensing, storage, power management, readout, and wireless communication. This review examines this coordination through two connected perspectives. Integration architecture describes how the harvesting and sensing units are physically arranged and functionally coupled, whereas signal–power matching describes how the temporal and electrical characteristics of harvested energy are aligned with the workloads required for system operation. The architectural discussion considers co‐packaged, monolithically integrated, and co‐functional systems, while the operating discussion considers always‐on, event‐driven, and multi‐modal sensing according to their dominant power requirements. Across these operating modes, the dominant matching condition shifts from average‐power delivery to burst‐energy management and shared‐load coordination. Connecting these structural and operational perspectives provides a framework for relating device configuration to energy use across the sensing system.

## 2 Energy Harvesting Devices: Fundamentals and Hybridization

**FIGURE 1:**
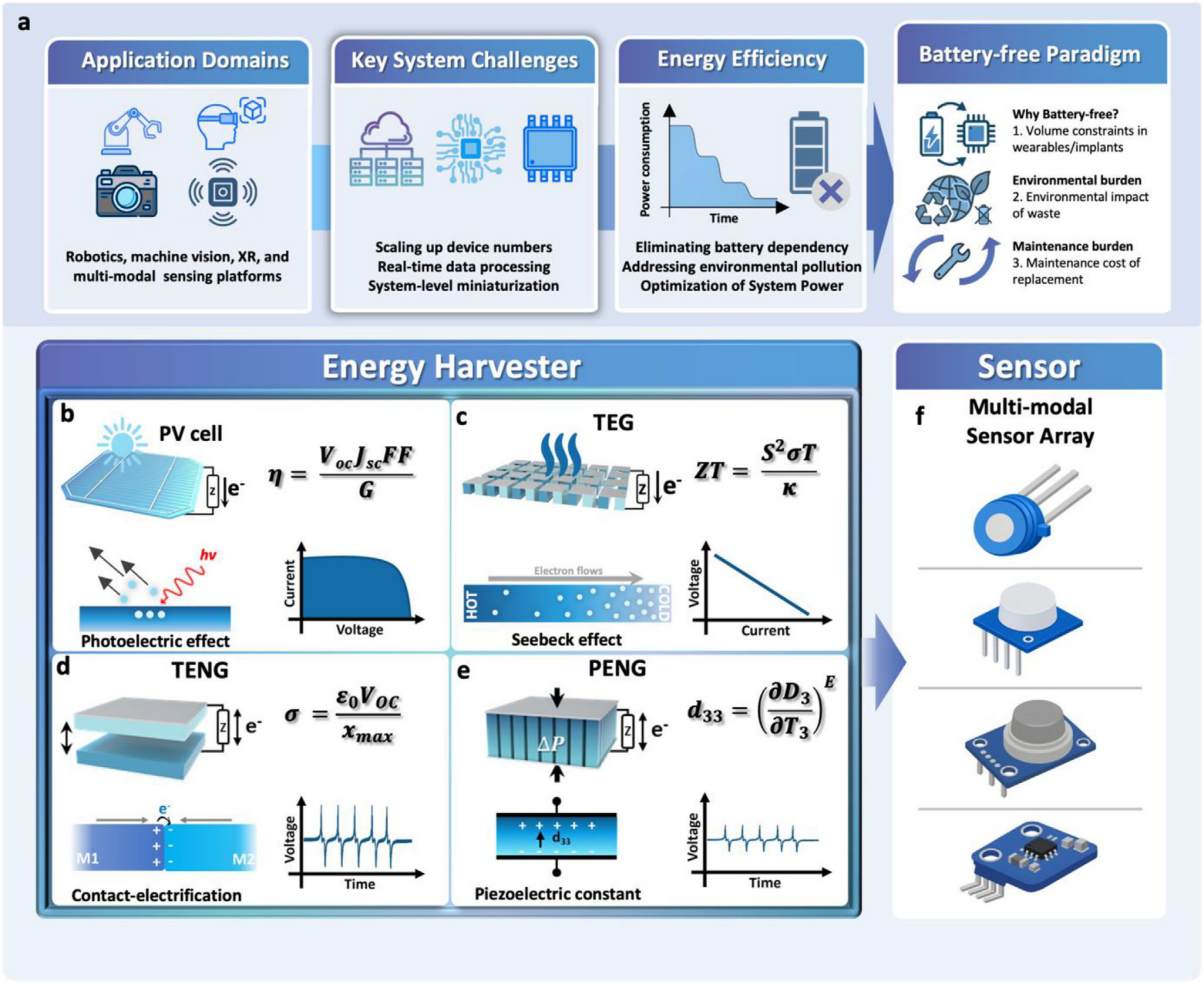
Fundamentals of EH technologies and the battery‐free sensing paradigm. (a) Overview of the transition toward battery‐free sensing systems, including representative application domains, system‐level challenges, and energy‐efficiency considerations. Working principles, typical electrical outputs, and governing equations of representative energy harvesters: (b) photovoltaic (PV) cells operating via the photoelectric effect, (c) thermoelectric generators (TEGs) based on the Seebeck effect, (d) triboelectric nanogenerators (TENGs) driven by contact electrification, and (e) piezoelectric nanogenerators (PENGs) utilizing the direct piezoelectric effect. (f) Representative sensor modalities for EH‐integrated sensing systems.

To realize battery‐free, self‐sustaining sensing platforms capable of continuous and autonomous operation across diverse real‐world environments, a thorough understanding of the individual EH devices that form the building blocks of such systems is essential [46, 47, 48] (Figure 1a). Among the most widely studied candidates, PV cells, TEGs, TENGs, and PENGs each transduce a distinct form of ambient energy into electricity through fundamentally different physical mechanisms. Their respective working principles, governing parameters, output characteristics, and recent research directions are introduced below to establish a solid foundation for the sensor‐integrated hybrid approaches addressed in subsequent sections.

The PV cell converts incident photon energy into direct current electricity through the photoelectric effect (Figure 1b). A conventional silicon‐based PV cell consists of a p‐n junction formed between a p‐type and an n‐type semiconductor layer, with metal electrodes deposited on the top and bottom surfaces. When photons with energies exceeding the semiconductor bandgap are absorbed, electron‐hole pairs are generated and immediately separated by the built‐in electric field at the junction, causing electrons to accumulate at the n‐side electrode and holes at the p‐side electrode, driving a continuous DC through the external circuit. The overall energy conversion performance is quantified by power conversion efficiency (PCE)

$$\eta =\frac{V_{OC\;}\times \;J_{SC}\;\times \;FF}{G}$$

where *Voc* is the open‐circuit voltage, *Jsc* is the short‐circuit current density, *FF* is the fill factor representing the rectangularity of the measured J‐V curve, and *G* is the incident irradiance [49, 50]. A conventional silicon single‐junction cell typically delivers a Voc near 0.6 V and a PCE of 15% to 27% under AM1.5G conditions [51, 52, 53]. Recent research has increasingly focused on perovskite absorber‐based devices as a practical route to overcome the efficiency ceiling of single‐junction silicon solar cells [54, 55, 56].

The TEG converts spatial temperature gradients directly into DC electricity through the Seebeck effect (Figure 1c). A standard TEG module consists of multiple p‐type and n‐type thermoelectric legs connected thermally in parallel and electrically in series between two ceramic plates. When a temperature difference ΔT is maintained across the device, thermally energized carriers in each leg diffuse toward the cold side, establishing an internal potential difference. The open‐circuit output voltage is expressed as

$$\;V_{OC}=S\;\times \;\Delta T$$

where *S* is the Seebeck coefficient of the thermoelectric material [57]. The intrinsic material quality is captured by the dimensionless figure of merit

$$ZT=\frac{S^{2}\;\times \;\sigma \;\times T}{\kappa }$$

where *σ* is the electrical conductivity, *T* is the mean absolute temperature, and *κ* is the thermal conductivity [58]. Because S, σ, and κ are physically coupled in most materials, simultaneously optimizing all three presents the central design challenge in thermoelectrics. If early research efforts primarily focused on improving the figure of merit (*ZT*) at the materials level through doping and band structure engineering, more recent studies have shifted attention toward the structural design of the device architecture itself [59, 60].

The TENG generates electricity by coupling contact electrification and electrostatic induction (Figure 1d). In the contact‐separation mode, two films composed of materials with different triboelectric polarities undergo periodic contact and separation. Upon contact, charge transfer across the dielectric interface leaves opposite surface charge densities on each film. As the films separate, the resulting charge imbalance induces a time‐varying potential difference between the back electrodes, driving an alternating current through the external circuit. The fundamental relationship between the triboelectric surface charge density and the open‐circuit voltage is expressed as

$$\sigma =\frac{\varepsilon_{0}\;\times \;V_{OC}}{x_{max}}$$

where *ε_0_* is the vacuum permittivity, and *x_max_* is the maximum separation distance between the two triboelectric layers [61, 62]. The AC output of TENGs is characterized by sharp spike‐shaped voltage pulses with Voc values typically ranging from tens to several hundreds of volts, reflecting the capacitive nature of the device [63, 64, 65, 66, 67]. The electrical output can also be adjusted through electrode‐level output management and interfacial charge‐transfer engineering, providing additional control over the voltage and transferred charge characteristics of TENGs [68, 69]. The field of TENGs has progressively shifted from output enhancement under low‐frequency mechanical excitation toward wireless power delivery via high‐frequency ultrasound, reflecting a transition in target applications from wearable energy harvesters to implantable medical devices [70, 71, 72].

The PENG converts mechanically induced stress into electricity through the direct piezoelectric effect (Figure 1e). When an external force is applied to a polar crystal, the unit cell deforms, and the centers of positive and negative charge are spatially displaced, breaking centrosymmetry and generating a macroscopic polarization. The resulting surface polarization charges drive a transient current through the external circuit proportional to the rate of change of applied stress. The intrinsic piezoelectric sensitivity along the poling axis is described by the piezoelectric strain coefficient

$$\;d_{33}={\left(\frac{\partial D_{3}}{\partial T_{3}}\right)}^{E}$$

where *D_3_* is the electric displacement along the poling direction, *T_3_* is the applied mechanical stress, and the superscript *E* denotes measurement under constant electric field [73]. PENGs produce AC voltage outputs consisting of symmetric bipolar pulses correlated with the direction and timing of the applied force [74]. One of the prominent research directions in PENGs is the fabrication of PVDF‐based nanofiber membranes via electrospinning, where simultaneous mechanical elongation and high‐voltage electric fields induce in situ β‐phase crystallization without a separate post‐poling step [75, 76, 77].

As reviewed above, PV cells, TEGs, TENGs, and PENGs have advanced considerably in output performance, mechanical flexibility, and operational stability through recent advances in materials and device engineering. Table 1 compares their representative electrical output characteristics, including output type, temporal behavior, voltage scale, and power‐density range. PV and TEG devices generally provide relatively continuous DC outputs under sustained illumination or temperature gradients, whereas TENG and PENG devices commonly generate intermittent or pulse‐like AC outputs under mechanical excitation. These differences influence the requirements for voltage regulation, impedance matching, rectification, and energy storage before the harvested output is delivered to the sensing system (Figure 1f). The output magnitude, source impedance, and temporal behavior also set electrical constraints for the integration architectures discussed in Section 3 and the signal–power matching strategies discussed in Section 4. Nevertheless, each device is inherently limited to a specific form of ambient energy, which constrains its applicability in real‐world environments where available energy sources vary continuously. The following section discusses hybrid EH systems that address this limitation by integrating complementary transduction mechanisms within a single platform.

**TABLE 1:** Representative electrical output characteristics of major energy‐harvesting technologies relevant to sensor integration.

Harvester	Ambient energy source	Output type	Temporal behavior	Representative voltage level	Representative power density^*^	Main power‐conditioning consideration	Refs.
PV cell	Incident photon energy	DC	Relatively continuous under sustained illumination	∼1 V per unit	10–100 µW cm^−2^ (indoor); 10–30 mW cm^−2^ (outdoor)	Voltage regulation and storage buffering under varying illumination	[31, 54, 55, 56]
TEG	Spatial temperature gradient	DC	Relatively continuous while a thermal gradient is maintained	∼mV scale per unit	10–100 µW cm^−2^ (wearable); 10–1000 mW cm^−2^ (industrial)	Low‐voltage boosting, impedance matching, and thermal‐interface management	[58, 59, 60]
TENG	Contact–separation or periodic mechanical motion	AC	Intermittent, sharp pulse‐like output	∼10^2^ V scale per unit	1–10 µW cm^−2^ (low‐frequency); 1–10 mW cm^−2^ (high‐frequency)	Rectification, charge management, and temporary energy storage	[61, 62, 63, 64, 65, 66, 67, 70, 71, 72]
PENG	Mechanically induced stress or deformation	AC	Intermittent bipolar pulses correlated with deformation	∼10^1^ V scale per unit	0.1–1 µW cm^−2^ (low‐stress); 1–10 mW cm^−2^ (high‐stress)	Rectification and buffer storage for transient mechanical inputs	[73, 74, 75, 76, 77]

* The reported voltage and power‐density ranges are representative values obtained under different device geometries and operating conditions and are intended to indicate characteristic output scales rather than provide a direct performance ranking among harvesting mechanisms.

### 2.1 Hybrid Energy Harvesting Devices

**FIGURE 2:**
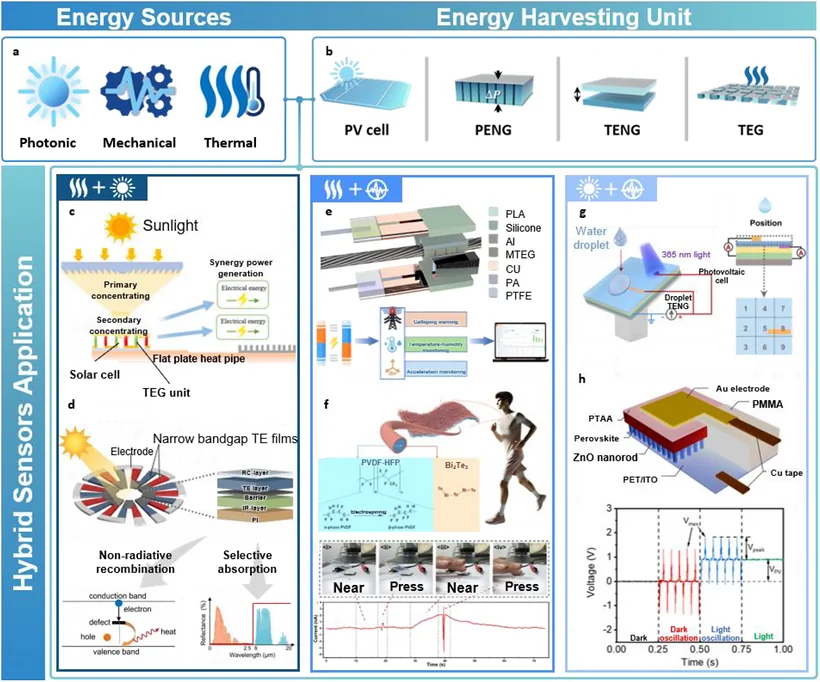
Hybrid EH systems combining complementary energy sources. (a) Representative ambient energy sources and (b) corresponding EH units used for hybrid energy harvesting. Thermal PV hybrids include (c) a concentrated solar TEG platform. Reproduced with permission from [80]. Copyright 2025 Elsevier. (d) A narrow bandgap selective absorption thermoelectric film. Reproduced with permission from [81]. Copyright 2025 Elsevier. Mechanical thermal hybrids include (e) an MTEG TENG device for transmission line monitoring. Reproduced with permission from [82]. Copyright 2024 IOP Publishing. (f) A flexible PVDF HFP Bi_2_Te_3_ core shell nanofiber film for wearable pressure and temperature sensing. Reproduced with permission from [83]. Copyright 2025 John Wiley and Sons. Mechanical PV hybrids include (g) a droplet TENG coupled with a PV cell. Reproduced with permission from [85]. Copyright 2023 Elsevier. (h) A ZnO nanorod PENG integrated with a perovskite solar cell. Reproduced under the terms of the CC BY 4.0 license from [87]. Copyright 2025 The Authors, published by Elsevier.

Single source EH systems face fundamental limitations in delivering continuous power under fluctuating real‐world conditions, as each transduction mechanism responds only to a specific ambient energy form. Hybridization can also combine complementary transduction mechanisms driven by the same mechanical input, allowing different components of deformation, contact, or motion to contribute to the electrical output [78]. To address this, hybrid devices integrating two or more complementary conversion mechanisms have been developed and are broadly categorized into mechanical‐PV, mechanical‐thermal, and thermal‐PV configurations (Figure 2a,b).

Thermal+PV hybrid systems exploit the complementary spectral responses of solar cells and TEGs to support power generation across varying illumination conditions. In 2024, Tan and Chan developed a PV‐TEG hybrid that uses radiative cooling of PV panels at night to sustain a temperature differential across TEG modules; two series‐connected 4 × 4 cm TEGs produced a peak output of 7.7 mW at a temperature difference of 5°C, demonstrating continuous 24‐h power generation for low‐power household applications [79]. Under concentrated solar conditions, in 2025, Wang et al. demonstrated a concentrator photovoltaic system integrating a TEG with a flat heat pipe (FHP) (Figure 2c) in which placing the TEG at the FHP evaporator section improved TEG output power by 230% to 361% and raised PV efficiency from 7.84% to 9.41% as the FHP bending angle increased from 0° to 90°, revealing that thermal interface design governs overall system performance in concentrated Thermal + PV architectures [80]. Hou et al. further unified the photothermal and thermoelectric functions within a single Ag_2_Se thin film, where an Ag/W infrared‐reflective base layer and an Al_2_O_3_/SiO_2_ dual antireflection coating raised the photothermal conversion efficiency to 87.6% and yielded a maximum power density of 0.17 W cm^−2^ under 1‐sun irradiation, with a stable outdoor peak output of 1 W (Figure 2d) [81].

Mech+Thermal hybrid systems are well suited for wearable electronics and infrastructure monitoring, where mechanical and thermal energy coexist. In 2024, Feng et al. developed a micro‐TEG (MTEG)‐TENG prototype for transmission line health monitoring that harvested both vibrational and Joule‐heating energy, achieving a TENG open‐circuit voltage of 80.3 V and an MTEG output of 1.094 V with a 5.36% improvement in total EH performance over a single‐source TEG system (Figure 2e) [82]. In 2025, Li et al. fabricated a flexible PVDF‐HFP/Bi_2_Te_3_ core‐shell piezoelectric‐thermoelectric coupled nanofiber film via electrospinning and magnetron sputtering, producing a piezoelectric output of 70 V at 5 N and a Seebeck coefficient of 77.4 µV K^−1^ at 320 K for simultaneous skin pressure and temperature monitoring (Figure 2f) [83]. Complementing these approaches, Huang et al. coupled TEG and TENG outputs to a neuromorphic oxide transistor, realizing tactile‐thermal dual‐modal sensing with TEG outputs ranging from 0.40 to 0.75 V for temperature differentials between 18.6 and 28.5°C [84].

Mech+PV hybrid systems harvest mechanical and solar energy simultaneously through shared‐electrode or multilayer designs. In 2022, Bao et al. demonstrated a PV cell‐TENG device with a common LaNiO_3_ bottom electrode that concurrently scavenged light (365 nm LED, 396 mW cm^−2^) and liquid‐droplet mechanical energy, achieving a peak current of 16.96 µA and a maximum peak power density of 3.71 mW m^−2^ (Figure 2g) [85]. Extending this TENG‐based concept, Xie et al. fabricated a raindrop solar cell by integrating a MoO_3_ electron‐blocking layer‐based TENG (MT‐TENG) with a perovskite solar cell through a shared ITO electrode, where MoO_3_ insertion increased the surface charge density of the FEP triboelectric layer by 101.1 times; the resulting device achieved a raindrop mechanical energy conversion efficiency of 12.49% and an output power of 0.68 mW while sustaining a perovskite PCE of 19.38%, enabling a 2.2 µF capacitor to be charged to 5 V in 175 s [86]. These results demonstrate that shared‐electrode integration in TENG‐PV systems is a robust design principle across diverse material platforms. Beyond TENG‐based systems, Zhang et al. showed that PENG units can equally serve as the mechanical harvesting component by integrating ZnO nanorods with a perovskite solar cell; the resulting hybrid exploited the piezo‐phototronic effect to modulate PV output under oscillation, achieving a PCE of 7.37% with a piezoelectric peak voltage of 1.36 V in the dark and 0.94 V under 1‐sun illumination (Figure 2h) [87].

These hybrid‐EH systems demonstrate that integrating complementary conversion mechanisms into a single device platform is a viable strategy for overcoming the output discontinuity inherent in single‐source approaches [88, 89]. Across all three categories, the key design principle is to carefully engineer structural‐ or material‐level interfaces so that each conversion mechanism can operate independently with minimal mutual interference. Future efforts should therefore be directed toward multi‐source architectures that combine three or more conversion mechanisms, coupled with on‐board power management circuits and energy storage components, with the ultimate goal of realizing fully self‐sustaining systems. These advances provide a critical foundation for sensor‐integrated hybrid harvesters, where energy generation and signal acquisition are inherently unified into a single, co‐designed system.

## 3 Energy Harvester‐Sensor‐Integrated Approach

**FIGURE 3:**
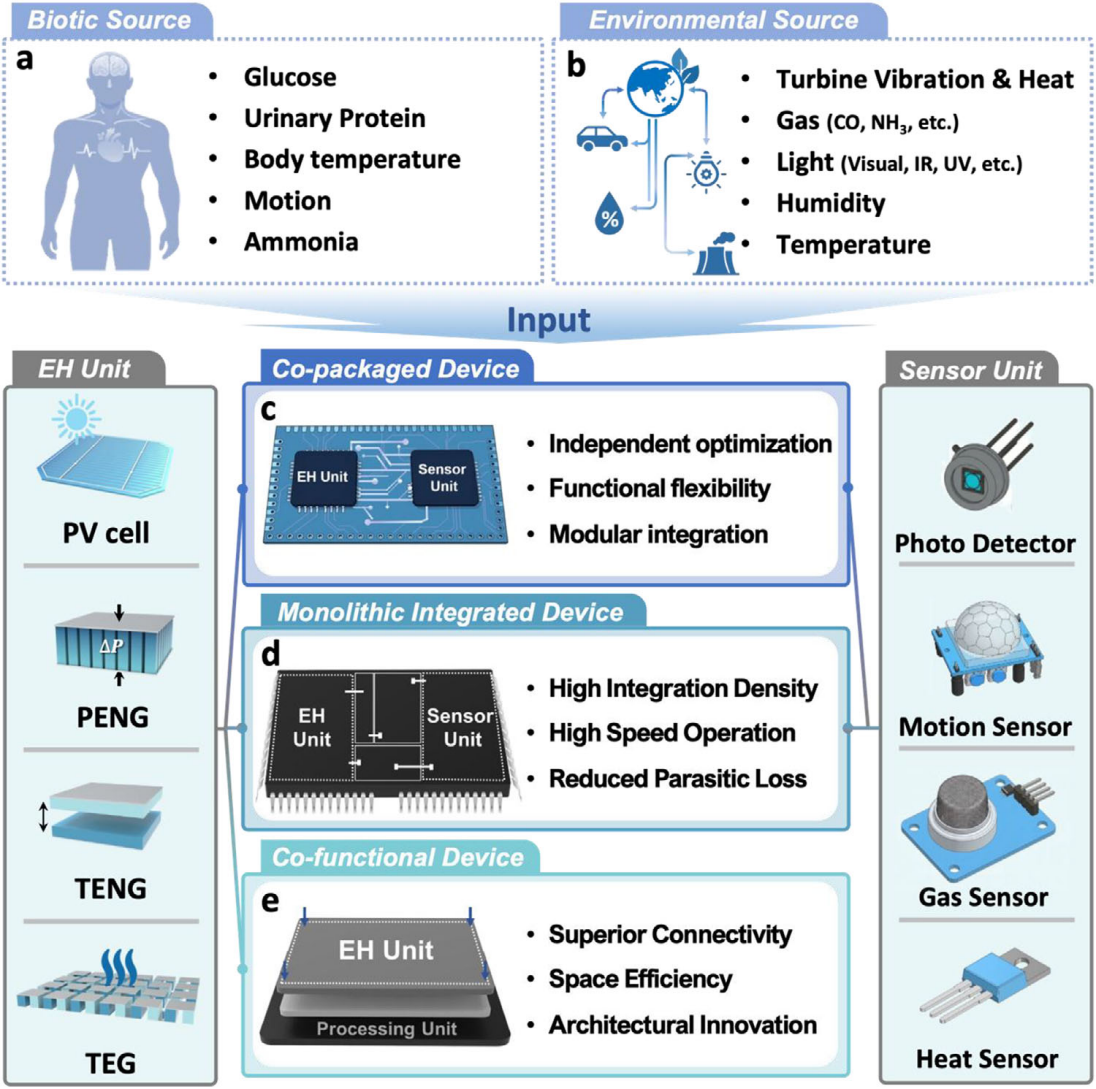
Schematic classification of EH‐integrated sensor systems according to their device architecture. Ambient input sources are categorized as (a) biotic and (b) environmental. Three integration strategies are illustrated: (c) co‐packaged, (d) monolithically integrated, and (e) co‐functional devices.

The integration of EH units with sensing capabilities represents a pivotal step toward fully autonomous wireless monitoring systems, where continuous power supply and real‐time signal acquisition are achieved within a single compact platform. As illustrated in Figure 3, the input energy sources sustaining such systems span two broad categories: biological and environmental. Biological sources originate from the human body and physiological processes, encompassing biochemical inputs such as glucose and urinary proteins as well as physical manifestations including body temperature, motion, and ammonia emissions (Figure 3a). Environmental sources cover a wide range of ambient stimuli, including turbine vibration and associated waste heat, chemical gases such as CO and NH_3_, light across the visual, infrared, and ultraviolet spectra, ambient humidity, and temperature gradients (Figure 3b). Beyond utilizing these ambient energy sources, the advanced multifunctional sensing units within these integrated platforms can be specifically engineered to target non‐energetic chemical analytes. This facilitates the self‐powered monitoring of hazardous gases, heavy metal ions, and regulated substances, thereby expanding the system's utility toward security and public health surveillance. These input signals not only power the onboard generation system but also carry the critical physical or chemical information targeted for detection, thereby providing a logical foundation for the functional co‐design of the EH and sensing subsystems.

Given this inherent correlation between the energy input and sensing target, the critical design challenge lies in the physical and functional orchestration of the EH and sensor units within a shared platform. The physical and functional relationship between the two units determines whether they remain independently assembled modules, are integrated as distinct functions within a shared device platform, or are merged through a common transduction pathway. The three architectures can be distinguished by the level at which the EH and sensing units are combined. In co‐packaged devices, independently fabricated and functionally distinct EH and sensing units are assembled within a shared package, preserving independent optimization and modular configurability (Figure 3c). In monolithically integrated devices, the two units are formed on a shared substrate or through a compatible fabrication process while retaining distinct energy‐conversion and sensing functions, which shortens the internal electrical pathway between them (Figure 3d). In co‐functional devices, the same active component or transduction pathway participates in both energy conversion and sensing, allowing the target stimulus to modulate the generated electrical output (Figure 3e). These three architectures provide the structural basis for EH‐integrated sensing systems, while the following sections examine how each architecture addresses different constraints in system‐level implementation.

### 3.1 Co‐Packaged Devices

**FIGURE 4:**
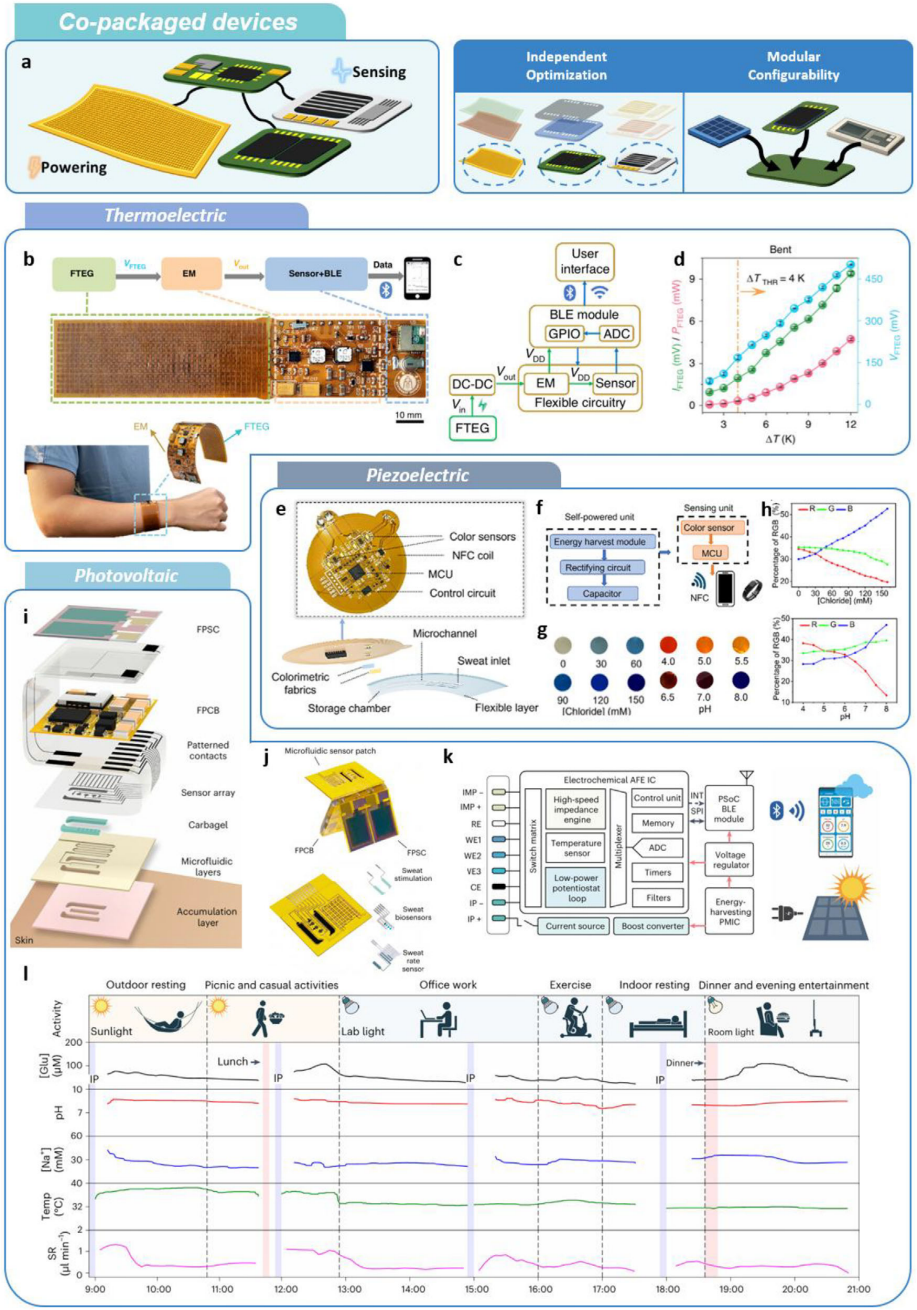
Co‐packaged devices for self‐powered sensing systems. (a) Schematic of co‐packaged devices and their key characteristics: independent optimization and modular configurability. (b) Body‐heat‐powered thermoelectric wearable system integrating an FTEG, EM circuit, and sensor/BLE module. (c) Circuit configuration showing thermoelectric energy delivery from the FTEG to the EM circuit, sensor, BLE module, and user interface. (d) FTEG output voltage, current, and power as a function of temperature difference. Adapted under the terms of the CC‐BY 4.0 license from [90]. Copyright 2023 Springer Nature. (e) Structural design of a piezoelectric self‐powered wireless sweat‐analysis patch. (f) Power and signal‐flow diagram showing the separated self‐powered unit and sweat‐sensing/readout unit. (g) Colorimetric responses of sensing fabrics to chloride concentration and pH. (h) RGB analysis of the colorimetric responses for chloride and pH detection. Adapted with permission from [91]. Copyright 2024 Elsevier. (i) Exploded view of the multilayer device assembly, including the FPSC, FPCB, sensor array, microfluidic layers, and sweat‐accumulation layer. (j) Photovoltaic‐powered wearable biosensing platform composed of a disposable sensor patch, FPCB, and FPSC. (k) System architecture connecting the FPSC, power‐management circuit, electrochemical sensing IC, BLE module, and signal‐processing components. (l) Continuous on‐body monitoring of glucose, pH, Na^+^, sweat rate, and temperature under different illumination and activity conditions. Adapted with permission [37]. Copyright 2023 Springer Nature.

Co‐packaged devices integrate energy‐harvesting, sensing, power‐management, storage, and readout units through modular assembly at the package or module level. Each unit remains a distinct functional block, allowing the platform to combine heterogeneous components without forcing them into a single fabrication process. As shown in Figure 4a, co‐packaged integration is based on independent optimization and modular configurability. The harvester can be specifically tailored to convert energy from a targeted ambient source, while the sensor and readout blocks can be designed for selectivity, sample handling, signal acquisition, and wireless communication. This modular route is useful for wearable and self‐powered sensing systems, where the energy source, sensing target, and replacement cycle often impose different constraints on each component. The harvester‐sensor combination can also be changed when the target or energy source changes, without requiring redesign of the entire platform.

The advantage of modular assembly becomes clear when the harvester, power‐conditioning circuit, and sensing/readout block operate under different electrical requirements. Yang et al. reported a body‐heat‐powered wearable thermoelectric system that combines a flexible thermoelectric generator (TEG), energy‐management electronics, and a sensor/Bluetooth low energy (BLE) module, as shown in Figure 4b–d [90]. The TEG harvests low‐grade body heat, while the DC‐DC converter and energy‐management circuit boost the low‐voltage output into a usable supply for the sensor and BLE module. The system operated under a temperature difference of approximately 4 K, recharged from an input voltage of 30 mV, and completed BLE data transmission in approximately 1.6 s. In this modular configuration, energy harvesting, power conditioning, and wireless sensing remain separately optimized while being connected into a body‐heat‐powered monitoring platform.

Sweat analysis introduces a different integration challenge because the energy harvester and biochemical sensor operate under different mechanical and fluidic requirements. A piezoelectric harvester relies on repeated deformation to generate electrical charge, whereas a microfluidic sweat sensor requires conformal skin contact, stable sweat collection, and controlled fluid transport. Cao et al. addressed these requirements using a self‐powered wireless patch in which the piezoelectric unit is physically separated from the sweat‐sensing module, as shown in Figure 4e–h [91]. Driven by human motion, the piezoelectric unit supplies power through a rectifying circuit and a capacitor to operate the system's microcontroller unit (MCU) and near‐field communication (NFC) coil. Human motion induces mechanical deformation in the piezoelectric unit, and the generated electrical energy is transferred via a rectifying circuit and temporary storage capacitor to power the MCU and NFC coil. The sensing unit collects sweat through a microchannel, analyzes chloride concentration and pH using colorimetric fabrics, and converts the color changes into RGB signals. Co‐packaging allows mechanical energy harvesting and biochemical sweat analysis to remain separately optimized while operating within a single wearable patch.

Co‐packaging is also useful when components in a wearable system have different lifetimes and replacement cycles. Min et al. reported a photovoltaic‐powered autonomous biosensor in which the reusable energy and readout modules are physically separated from a disposable sweat‐sensing patch, as shown in Figure 4i–l [37]. The platform combines a flexible perovskite solar cell, a flexible printed circuit board containing a power‐management unit, an electrochemical sensing IC, a Bluetooth module, and a layered microfluidic sweat sensor. The photovoltaic and readout modules powered the monitoring of glucose, pH, Na+, sweat rate, and temperature under different illumination and activity conditions. Because the microfluidic sensor directly contacts skin and biofluids, periodic replacement may be needed due to contamination, biofouling, reagent degradation, or hygiene concerns. Conversely, the photovoltaic and readout electronics maintain long‐term reusability. Physical separation between these parts allows the sweat‐sensing patch to be replaced while retaining the photovoltaic and electronic modules.

The thermoelectric, piezoelectric, and PV systems discussed above use different energy sources and sensing modalities, but they share a modular integration scheme. Co‐packaging allows low‐voltage thermoelectric output to be conditioned before wireless readout, separates mechanical power generation from biochemical sweat analysis, and divides reusable electronics from replaceable biofluid‐contacting patches. This architecture is useful when heterogeneous components require different materials, operating environments, or maintenance cycles that are difficult to reconcile within a single fabrication process. By preserving the functional independence of each module, co‐packaged integration provides flexibility in selecting, replacing, or reconfiguring specific components according to the energy source and sensing target. The same modularity, however, also leaves physical interconnections between separately assembled blocks, making the length and stability of the power and signal pathways important design considerations. Consequently, an alternative integration strategy is required to minimize these geometric losses by forming the harvesting and sensing units on a shared substrate or within a compatible process.

### 3.2 Monolithically Integrated Devices

**FIGURE 5:**
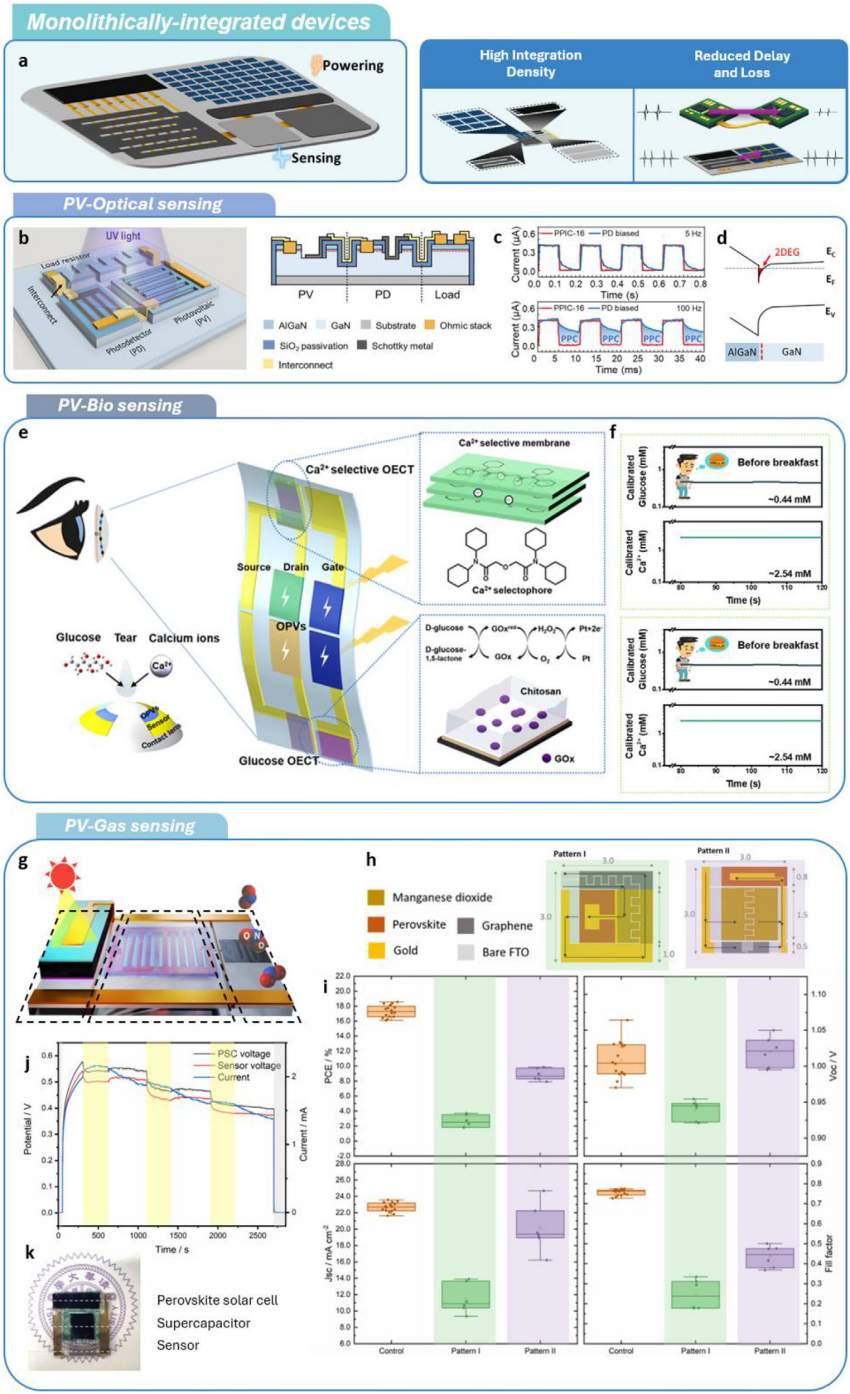
Monolithically integrated devices for self‐powered sensing systems. (a) Schematic of monolithically integrated devices and their key characteristics: high integration density and reduced delay/loss through shortened electrical pathways. (b) On‐chip AlGaN/GaN photovoltaic‐photodetector integrated configuration (PPIC) integrating a PV unit, PD unit, and load resistor for self‐powered UV sensing. (c) Transient response comparison between the PPIC and externally biased PD, showing suppressed persistent photoconductivity and improved response speed. (d) Energy band diagram of the AlGaN/GaN heterojunction showing the 2DEG‐based on‐chip transport pathway. Adapted under the terms of the CC‐BY license from [93]. Copyright 2025 Wiley‐VCH. (e) Flexible organic integrated platform combining OSCs and OECT‐based glucose/Ca^2+^ sensors for self‐powered ocular monitoring. (f) Calibrated responses of self‐powered OECT sensors to glucose and Ca^2+^ concentration changes. Adapted under the terms of the CC‐BY 4.0 license from [94]. Copyright 2022 Springer Nature. (g) Three‐in‐one monolithic NO_2_ sensing platform integrating a PSC, MnSC, and GnSR on a single glass substrate. (h) Pattern designs of the integrated PSC for layout‐level optimization. (i) Photovoltaic performance of the patterned PSCs, including PCE, V_oc_, J_sc_, and fill factor. (j) Integrated sensing response of the PSC‐MnSC‐GnSR platform under light irradiation and NO_2_ exposure. (k) Optical image of the fabricated 3‐in‐1 integrated chip. Adapted under the terms of the CC‐BY 4.0 license from [95]. Copyright 2025 American Chemical Society.

Monolithically integrated devices represent a device‐level architecture in which energy‐harvesting and sensing units, and in some cases storage, power‐management, or readout components, are formed on a shared substrate or within a compatible process [92]. In contrast to co‐packaged systems, where functional modules are separately optimized and assembled, monolithic integration reduces the physical distance and electrical pathway between harvesting and sensing units [93]. As shown in Figure 5a, this architecture places the functional blocks within a common platform, allowing the generated voltage or current to be delivered to the sensor through shorter interconnection paths. Shortened pathways can reduce parasitic resistance, capacitance, signal loss, and response delay, while helping the harvested output match the sensor operating conditions. The design value of monolithic integration lies in this closer connection between energy conversion and sensing operation, which can support direct bias coupling, operation‐point matching, or harvesting‐storage‐sensing chains depending on the sensing target.

Shortened electrical pathways are particularly useful when the harvested voltage can be delivered directly to the sensor as an operating bias. Pu and Liang demonstrated this concept through an AlGaN/GaN‐based photovoltaic‐photodetector (PV‐PD) integrated circuit for UV optical sensing, as shown in Figure 5b–d [93]. This on‐chip platform integrates a photovoltaic unit, a Schottky‐type photodetector unit, and a load resistor on a single AlGaN/GaN‐on‐Si substrate. The photovoltaic unit absorbs UV light below 365 nm and generates a photovoltage that directly biases the photodetector, eliminating the need for an external power source. In this monolithic design, the harvesting unit does more than supply continuous power; it synchronizes the bias field with the incident UV illumination. When light is present, the photovoltage drives photocarrier collection. When the light is turned off, the bias field instantly disappears, halting the collection of residual photocarriers. This synchronized operation effectively suppresses persistent photoconductivity and accelerates the response speed compared to conventionally biased devices. The integrated circuit exhibited a peak responsivity of 19.2 A W^−1^, a detectivity of 9.3 × 10^13^ Jones, rise and fall times of 61.18 and 97.79 µs, respectively, and a −3 dB bandwidth of 6230 Hz. The two‐dimensional electron gas (2DEG) formed at the AlGaN/GaN heterojunction underpins this high‐speed operation by acting as a highly conductive internal pathway. This built‐in channel facilitates instantaneous bias delivery from the harvesting unit to the sensor, demonstrating that physically and electrically binding the energy conversion and sensing mechanisms improves the photodetection process.

While the preceding UV sensing example demonstrates the effect of monolithic integration on response speed, the following case illustrates how this architecture enables precise operation‐point matching. Lin et al. reported a self‐powered multiplexed ocular monitoring system in which organic solar cells (OSCs) and organic electrochemical transistor (OECT) sensors are fabricated on the same flexible substrate, as shown in Figure 5e,f [94]. In this monolithic platform, the OSCs do more than serve as a generic power source; they are specifically configured as inverted and conventional cells to supply the exact gate and drain biases required for OECT operation. This built‐in electrical matching eliminates the need for complex peripheral circuits. Driven directly by PV output, the OECT sensors accurately convert changes in biomarker concentrations into variations in drain current. For glucose sensing, a functionalized Pt gate induces an enzymatic reaction, causing the drain current to decrease from 19.5 to 17.2 µA as the glucose concentration increases from 0.1 to 1 mM. Similarly, for calcium sensing, an ion‐selective membrane modulates the channel current, decreasing it from 127.9 to 110.2 µA across a concentration range of 0.1 to 10 mM. By co‐designing the energy conversion and biochemical sensing units on a shared platform, this system establishes direct device‐level synchronization while significantly reducing the spatial footprint.

When the harvested input fluctuates over time, integrating energy storage into the same platform helps buffer the generated energy and form a harvesting‐storage‐sensing chain for stable sensor operation. He et al. demonstrated this concept through a monolithically integrated three‐in‐one NO_2_ sensing system, as shown in Figure 5g–k [95]. Fabricated on a single glass substrate, the platform consists of a perovskite solar cell (PSC), an MnO_2_‐based micro‐supercapacitor (MnSC), and a graphene nanoplatelet‐based gas sensor (GnSR). Unlike optical or biological systems, gas sensors demand a highly stable operating voltage, making them vulnerable to the continuous fluctuations of solar input. To address this variability, the integrated MnSC acts as an intermediate storage unit that buffers the energy generated by the PSC whose layout and current pathways were optimized to maximize overall device performance and supplies it steadily to the GnSR. Upon NO_2_ exposure, the resistance of the polyvinylpyrrolidone‐graphene sensing layer changes, resulting in measurable variations in the sensor and PSC voltages. The integrated device exhibited a photovoltaic conversion efficiency of 8.84%, a supercapacitor energy density of 0.76 µWh cm^−2^, and a gas response of 10.8% at 10 ppm NO_2_. By physically and electrically connecting these three distinct functions, this monolithic architecture transforms the device from a simple coupled sensor into a comprehensive self‐powered energy management platform.

Across these cases, monolithic integration reduces the physical and electrical separation between energy harvesting and sensing functions. The PV‐PD circuit uses the photovoltaic output as a local bias, the OSC‐OECT ocular sensor matches photovoltaic outputs to the operating points of biomarker sensors, and the PSC‐MnSC‐GnSR system extends the energy path through storage before gas sensing. These architectures connect harvested output to sensor operation through shortened pathways, local biasing, and storage‐linked supply. In many monolithic systems, harvesting and sensing still remain distinguishable functions. For highly compact systems or stimuli that directly perturb the energy‐conversion process, the two roles can be merged within a single functional device, allowing the generated electrical output to serve as both power and sensing signal.

### 3.3 Co‐Functional Devices

**FIGURE 6:**
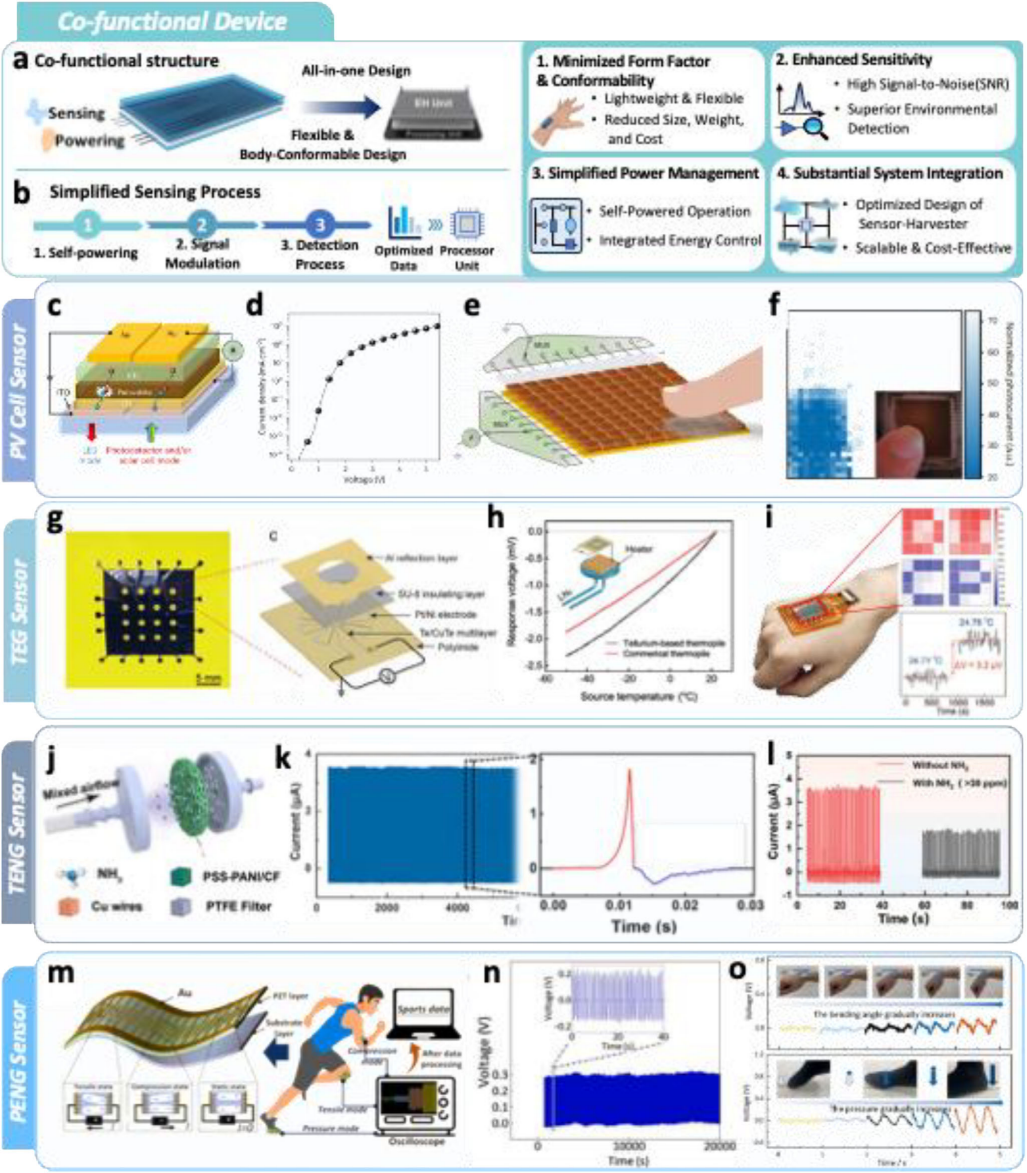
Co‐functional device architecture and representative demonstrations for each energy‐harvesting mechanism. (a) Schematic of all‐in‐one co‐functional structure. (b) Three step operational flow from self‐powering to signal modulation and detection output, highlighting four key design advantages. For each mechanism, the panels follow a consistent sequence from device structure to EH performance and sensing capability. For the PV cell sensor, (c) device structure, (d) PV output characterization, and (e,f) spatially resolved multi‐modal sensing demonstrations. Reproduced under the terms of the CC‐BY license from [107]. Copyright 2024 The Authors, published by Springer Nature. For the TEG sensor, (g) flexible thermoelectric multilayer film structure, (h) Seebeck voltage response with respect to source temperature, and (i) wrist‐worn spatial temperature mapping. Reproduced with permission from [108]. Copyright 2024 John Wiley and Sons. For the TENG sensor, (j) airflow‐driven PSS‐PANI composite device structure, (k) stable electrical output characterization, and (l) output modulation upon NH_3_ exposure. Reproduced with permission from [111]. Copyright 2025 Elsevier. For the PENG sensor, (m) stacked multilayer piezoelectric nanofilm structure, (n) long‐term output stability, and (o) voltage responses to progressive bending angle and plantar pressure variations. Reproduced with permission from [114]. Copyright 2025 Elsevier.

Co‐functional devices represent the most compact integration strategy among the three architectures introduced in the preceding section. In this configuration, a single active component simultaneously performs EH and sensing without requiring a dedicated sensing layer. The energy harvester itself responds to changes in the surrounding environment and generates distinguishable electrical signals that carry sensory information, resulting in thin and lightweight devices well suited for body‐conformable and skin‐integrated applications (Figure 6a) [96, 97, 98, 99, 100].

The sensing process in co‐functional devices follows a unified operational sequence (Figure 6b). Under baseline conditions, the device continuously converts ambient energy, whether photonic, thermal, or mechanical, into a steady electrical output. When a target stimulus enters the energy conversion pathway, it directly modulates this baseline electrical response, producing a measurable change in current, voltage, or power density. The modulated signal is then processed by a detection unit and microcontroller to extract the target information. Because the harvesting process provides both operational energy and sensing signal from a single ambient input, the need for a separate sensing excitation or dedicated sensing layer can be reduced. This co‐functional design offers four principal advantages. First, the elimination of a separate sensing layer minimizes device thickness and weight, supporting flexible and body‐conformable designs with reduced fabrication cost [101, 102]. Second, because the sensing signal is derived directly from changes in the primary energy conversion process, background noise is inherently low, resulting in a high signal‐to‐noise ratio [103, 104]. Third, true self‐powered operation is achieved without reliance on external energy storage, as the device generates, senses, and transmits signals from the same ambient input [105]. Fourth, reduced component count and simplified interconnection lower fabrication complexity and support scalable deployment in practical monitoring systems [106].

When photonic energy serves as the ambient input, any perturbation in incident light directly modulates the electrical output of the harvesting layer, enabling simultaneous power generation and optical sensing from a single active material. As shown in Figure 6c, Bao et al. developed a perovskite light‐emitting diode array in which the same active perovskite layer functions either as a light source or as a PD depending on the applied bias direction [107]. This structural duality allows a single pixelated panel to perform both electroluminescence and photocurrent generation without any added sensor elements, directly realizing the co‐functional architecture. Figure 6d characterizes the EH performance of this panel. Under indoor illumination, the device achieves a specific detectivity of 6.08 × 10^1^ ^2^ Jones and a power conversion efficiency of 12.6%, confirming stable PV operation as the baseline for sensing. The high photoresponsivity across the visible range ensures that even small changes in incident light intensity produce measurable photocurrent variations, establishing the quantitative foundation for the sensing mode. Figure 6e illustrates the signal modulation mechanism. When an external stimulus interacts with the illuminated panel surface, the spatial distribution of incident intensity is locally altered, directly modulating the photocurrent output at the pixel level and producing a two‐dimensional signal map that encodes the spatial profile of the stimulus. The approximately linear relationship between illumination change and current response allows quantitative extraction of stimulus information without additional processing hardware. Figure 6f demonstrates the resulting application range. Operating through the identical pixelated structure used for EH, the panel supports touch localization, ambient illumination measurement, fingerprint pattern recognition, and photoplethysmography heart rate monitoring. Beyond display applications, Min et al. and Han et al. extended this PV co‐functional strategy to flexible perovskite solar cell nodes that simultaneously provide self‐sustaining power and serve as signal references for analyte‐induced current modulation in multiplexed biosensing platforms [37, 99].

With thermal energy acting as the primary ambient source, spatial temperature gradients drive thermoelectric conversion, and the resulting Seebeck voltage carries direct information about the thermal environment, enabling simultaneous EH and non‐contact temperature sensing. Figure 6g presents a flexible photothermoelectric detector developed by Guo et al. in which carbon‐based thermoelectric films are patterned on a flexible substrate [108]. The device converts incident infrared radiation into a local thermal gradient through photothermal absorption, and the Seebeck voltage generated across this gradient is measured directly as the sensing output without any external bias or reference circuit. Figure 6h characterizes the thermoelectric performance of the device. Across a source temperature range from minus 50°C to 110°C, the detector generates a well‐defined open‐circuit Seebeck voltage in the contactless measurement mode, exhibiting a sensitivity superior to that of commercial rigid thermopiles over the same range. The stable and reproducible voltage output confirms the device as a reliable thermoelectric energy converter, and the monotonic relationship between thermal gradient magnitude and Seebeck voltage provides the quantitative basis for the sensing modality in Figure 6i. Figure 6i shows the application of this capability to touchless thermosensation for electronic skin, enabling spatial mapping of the Seebeck voltage distribution across the detector area to reconstruct the thermal profile of a target surface with high spatial resolution. Beyond electronic skin, Chung et al. extended the thermal co‐functional concept to wearable health monitoring by using body heat‐driven thermoelectric harvesting to power a microneedle‐based sensor for continuous interstitial glucose monitoring [109], and Yu et al. demonstrated simultaneous thermal EH and fire warning through MXene‐integrated dual‐mode fibers [110].

With mechanical energy acting as the ambient driving source, both triboelectric and piezoelectric transduction mechanisms convert periodic deformation or contact motion into electrical output, and any change in the physical or chemical conditions at the active material surface produces a corresponding shift in that output, enabling co‐functional sensing without a separate sensing element. Figure 6j presents the PSS‐PANI/copper foam composite TENG developed by Wang et al., in which polyaniline is deposited onto copper foam electrodes as the chemically responsive surface layer [111]. The device is driven by high‐pressure pulsed airflow at an optimized driving pressure of 0.4 MPa, inducing repeated contact‐separation cycles between the composite electrodes. Figure 6k characterizes the EH performance and operational stability of this device. Under the prescribed airflow conditions, the device delivers an output current of 3.61 µA sufficient to power small electronic components, and the output signal remains stable in clean air over repeated actuation cycles. This stable baseline is essential for defining the measurable deviation that constitutes the sensing response. Figure 6l demonstrates the gas sensing capability derived from this baseline. When ammonia molecules interact with the polyaniline layer, the protonation of imine groups increases surface conductivity and modulates the charge transfer efficiency of the triboelectric process, systematically reducing the current output under identical mechanical excitation. The response recovers reversibly upon removal of the NH_3_ source and remains linear across the tested concentration range down to 100 ppm, enabling quantitative analyte monitoring from a single mechanically driven device structure. Beyond gas detection, Zarasvand et al. demonstrated TENG‐based monitoring of ice accretion and interfacial phase transitions on aircraft wings through triboelectric current variations [112], and Yu et al. developed an AI‐assisted droplet‐driven TENG for vancomycin concentration monitoring in which drug concentration directly modulates the triboelectric output [113].

Piezoelectric systems harvest mechanical energy through the direct piezoelectric effect and exhibit an equivalent co‐functional behavior when their electrical output is systematically modulated by specific environmental or physiological inputs. Figure 6m presents the self‐adhesive crystal‐enhanced multilayer nanofilm PENG developed by Song et al. [114], in which lead zirconate titanate (PZT)‐based piezoelectric layers are stacked onto a flexible substrate with gold electrodes and fabricated by electrospinning. The device is designed to respond to both tensile and compressive deformations, covering the full range of body motion states, and its electrical output is directly acquired by an oscilloscope for real‐time data processing without any intermediate signal conditioning circuit. Figure 6n characterizes the EH performance and long‐term operational stability of this device. During repeated cycles of body motion, the device delivers a stable voltage output for over 20 000 s, confirming that the stacked piezoelectric architecture maintains a consistent baseline output over extended use. This long‐term stability is critical for establishing the reproducible baseline from which physiological deviations can be quantitatively resolved. Figure 6o demonstrates the sensing capability built upon this stable baseline. In the upper panel, the output voltage increases systematically with gradually increasing bending angles from 15° to 75°, confirming that the piezoelectric response is sensitive to angular deformation in a discriminable and monotonic manner. In the lower panel, the device similarly resolves progressively increasing plantar pressure, with the output amplitude scaling proportionally to the applied load. These results establish that the same mechanical‐to‐electrical transduction process responsible for EH simultaneously encodes postural and pressure information, realizing co‐functional operation without any added sensing element. Zhao et al. designed a magnetically actuated piezoelectric device that combines piezoelectric nano‐FET biosensing with on‐demand contact‐mode switching, enabling spatially controlled cerebral cortex sensing driven by the piezoelectric output itself [115]. Hu et al. further applied piezoelectric vibration sensing to aircraft engine fault detection, where the vibrational signature of mechanical anomalies modulates the piezoelectric output in a manner that supports both energy recovery and structural health diagnosis [116]. Across these examples, TENG and PENG systems share the fundamental co‐functional principle that the mechanical energy input serves as both the power source and the information carrier, with environmental or physiological changes modifying the transduction pathway to yield a self‐powered sensing signal.

The co‐functional architecture represents a meaningful step toward fully autonomous sensing systems, as it resolves the conventional trade‐off between device simplicity and functional completeness by allowing a single active material to serve as both the power source and the sensing element. This structural convergence is particularly relevant in the context of wearable health monitoring and distributed IoT networks, where minimizing device footprint while maintaining continuous operation is a primary design constraint. Future development must focus on improving long‐term stability and overcoming cross‐sensitivity to ensure the reliable deployment of these systems. Ultimately, with the continuous expansion of material platforms, co‐functional devices will serve as the foundation for next‐generation self‐sustaining intelligent sensing architectures. Table 2 summarizes the structural characteristics of the three integration architectures discussed in Section 3. The comparison focuses on the harvester–sensor relationship, integration density, fabrication complexity, electrical path length, and component independence. Table 3 further summarizes the representative systems discussed in Section 3 according to their integration architecture, energy source, sensing target, power‐management or storage strategy, and key system‐level characteristics. These architectures establish different physical conditions for delivering harvested energy to the sensing function. Co‐packaged systems retain physically separated harvesting and sensing modules, monolithically integrated systems shorten the internal electrical pathways between distinct functions, and co‐functional systems couple energy conversion and sensing within the same transduction pathway. These structural differences provide the hardware basis for the signal–power matching strategies discussed in Section 4.

**TABLE 2:** Comparison of integration architectures for energy‐harvesting sensing systems.

Integration architecture	Harvester–sensor relationship	Integration density	Fabrication complexity	Electrical path length	Component independence	Refs.
Co‐packaged	Independently fabricated, functionally distinct units assembled within a shared package	Low	Low (Modular assembly)	Long (Inter‐module wiring)	High	[37, 90, 91]
Monolithically integrated	Distinct harvesting and sensing functions formed on a shared substrate or through a compatible process	Medium to high	High (Process compatibility required)	Short (On‐chip interconnection)	Moderate	[93, 94, 95]
Co‐functional	Same active component or transduction pathway participates in both energy conversion and sensing	High	Medium (Material‐ and mechanism‐dependent)	Minimal (Internal shared pathway)	Low	[107, 108, 111, 114]

**TABLE 3:** Representative EH‐integrated sensing systems classified by integration architecture.

Integration architecture	Energy source	Sensing target / modality	Power‐management / storage strategy	Key system metric / operation feature	Refs.
Co‐packaged	Body heat / TEG	Wearable sensing and BLE readout	DC‐DC conversion and energy‐management circuit	ΔT ≈ 4 K; recharge from 30 mV; BLE transmission ≈ 1.6 s	[90]
Co‐packaged	Human motion / PENG	Sweat Cl^−^ and pH	Rectification and temporary capacitor storage	Rectified PENG output operates MCU and NFC	[91]
Co‐packaged	Ambient light / PV	Glucose, pH, Na^+^, sweat rate, temperature	Power‐management unit + flexible PCB + BLE	Multi‐parameter on‐body monitoring under varying illumination/activity	[37]
Monolithically integrated	UV light / PV	UV photodetection	Direct local photovoltaic bias	19.2 A W^−1^; 9.3 × 10^13^ Jones; 61.18/97.79 µs; 6230 Hz	[93]
Monolithically integrated	Light / OSC	Glucose and Ca^2+^	Direct gate/drain bias supplied by OSCs	Glucose current 19.5→17.2 µA; Ca^2+^ 127.9→110.2 µA	[94]
Monolithically integrated	Light / PSC	NO_2_ gas	Integrated MnO_2_ micro‐supercapacitor	PCE 8.84%; 0.76 µWh cm^−2^; 10.8% response at 10 ppm NO_2_	[95]
Co‐functional	Light / PV	Optical, tactile, and physiological signals	Direct shared photoelectric transduction	6.08 × 10^12^ Jones; PCE 12.6%	[107]
Co‐functional	Thermal gradient / IR	Contactless temperature sensing	Direct Seebeck‐voltage readout	Source temperature range −50°C to 110°C	[108]
Co‐functional	Mechanical airflow / TENG	NH_3_ gas	Direct triboelectric output	3.61 µA output; sensing down to 100 ppm NH_3_	[111]
Co‐functional	Mechanical deformation / PENG	Bending and plantar pressure	Direct piezoelectric output	Stable output >20 000 s; bending angle 15°–75°	[114]

## 4 Signal‐Power Matching in EH‐Integrated Sensing Systems

Section 3 established the structural basis of EH‐integrated sensors by classifying them into co‐packaged, monolithically integrated, and co‐functional architectures. These architectures describe how harvesting and sensing functions are physically arranged and functionally coupled within a shared platform, while also shaping the design of storage, power‐management, and readout pathways. However, structural integration alone does not determine system operation. Once the harvesting and sensing functions are integrated, the system must be operated according to the temporal characteristics of the target signal and the available harvested‐energy profile.

Signal‐power matching refers to aligning the temporal and electrical characteristics of harvested energy with the workloads required for sensing, energy storage, data processing, and wireless communication. The matching condition depends on both the amount and timing of available energy because different system functions impose different power demands over time. Always‐on, event‐driven, and multi‐modal sensing are treated here as dominant operating paradigms rather than mutually exclusive device classes, and more than one mode can coexist within a practical system. Always‐on operation is primarily governed by average‐power matching, event‐driven operation by low‐standby readiness and burst‐energy delivery, and multi‐modal operation by shared‐load coordination across concurrent sensing channels. The integration strategy determines the available hardware configuration, whereas the operating paradigm determines how harvested energy is distributed over time to support sensing, processing, and communication.

### 4.1 Always‐on Sensing

**FIGURE 7:**
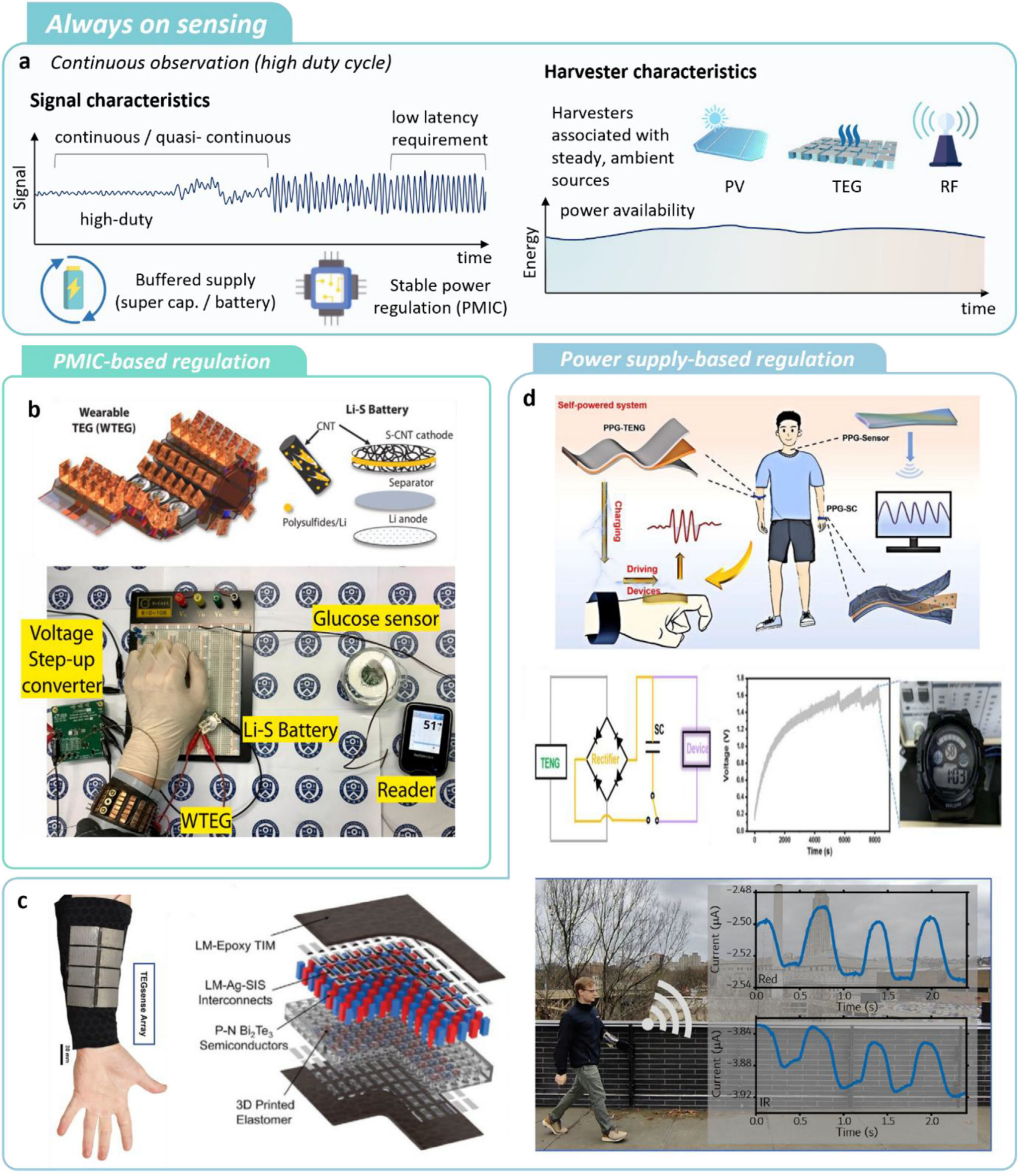
Always‐on sensing under harvested energy. (a) Schematic description of always‐on sensing as a P_avg_‐limited operating paradigm for continuous or quasi‐continuous signals. Stable or slowly varying energy sources, including PV, TEG, and RF harvesters, are combined with buffer storage and PMICs to support high‐duty sensing, readout, and communication. (b) Wearable TEG (WTEG) and Li‐S battery system for continuous glucose monitoring, where body heat is converted, boosted through a voltage step‐up converter, and stored in a Li‐S battery to operate a commercial glucose sensor. Adapted with permission from [117]. Copyright 2020 Elsevier. (c) Stretchable TEG‐based platform for body‐heat‐powered photoplethysmography (PPG) monitoring. The TEGsense system consists of a stretchable TEG array, capacitor bank, and low‐power PPG board, enabling harvested thermal energy to be boosted, stored, and periodically released for PPG acquisition and BLE transmission. Reproduced with permission from [118]. Copyright 2024 Wiley‐VCH GmbH. (d) TENG‐supercapacitor self‐charging system for storage‐assisted wearable operation, where biomechanical energy is harvested by a flexible TENG, stored in a supercapacitor, and supplied to wearable electronics or sensing modules when needed. Adapted with permission from [119]. Copyright 2025 Royal Society of Chemistry.

Always‐on sensing represents the average‐power‐limited operating mode of EH‐integrated sensors. In this mode, the target signal is continuous or quasi‐continuous, and the system must preserve sensing availability over extended periods. Physiological monitoring, environmental tracking, and structural health monitoring are typical cases in which information can be lost when the sampling interval becomes too long or when the supply voltage becomes unstable. As illustrated in Figure 7a, the design challenge lies in sustaining sensing, readout, and communication functionalities under harvested energy inputs characterized by stochastic magnitudes and intermittent timing. This average‐power matching can be achieved either by regulating weak harvested outputs through power management integrated circuits (PMICs) or by designing storage‐centered power supplies that buffer harvested energy and release it according to sensing demand.

In PMIC‐based regulation, the harvested output is first converted into a regulated operation voltage to safely power the sensing module. Kim et al. reported this strategy using a wearable thermoelectric generator (WTEG) integrated with a lithium‐sulfur (Li‐S) battery for continuous glucose monitoring, as shown in Figure 7b [117]. Body heat was harvested by the WTEG, boosted through a voltage step‐up converter, and stored in the Li‐S battery before powering a commercial glucose sensor. The WTEG delivered a maximum power output of 378 µW with an open‐circuit voltage of 63 mV under matched‐load conditions, corresponding to a power density of 8.68 µW cm^−2^. During glucose measurement, the commercial sensor consumed an average power of 64 µW, while the boosted WTEG output supplied an average power of 83 µW at approximately 2.16 V. The Li‐S battery buffered the difference between the fluctuating thermoelectric output and the time‐varying sensor load, allowing low‐grade body heat to be converted into a regulated supply for continuous sensing.

Storage‐centered power supplies mitigate the same average‐power constraints through the temporal redistribution of energy accumulation and release. Zadan et al. reported this approach using TEGsense, a stretchable TEG‐based wearable platform for body‐heat‐powered photoplethysmography monitoring, as shown in Figure 7c [118]. The system combines a forearm‐worn stretchable TEG array, an elastomer‐supported thermoelectric structure, a capacitor array, and a custom low‐power photonic PPG board. Parallel TEG connection reduced impedance mismatch with the boost converter, while a 10.8 mF low‐ESR capacitor bank shortened the charging time and supplied the surge current required for circuit activation. The harvested thermal energy was accumulated in the capacitor bank, and the PPG board was activated only when the stored energy reached the operating window. During each active period, the PPG sensor acquired red and infrared signals at 100 Hz for 3 s, followed by BLE transmission. In this configuration, the capacitor bank operates as a temporary power supply that converts continuous low‐level body‐heat harvesting into repeated sensing and communication windows.

The role of storage becomes more pronounced when the harvested energy comes from intermittent body motion rather than a relatively continuous thermal gradient. Because motion‐driven harvesting generates energy intermittently during discrete biomechanical activities, such as walking, tapping, or bending, the power management system must dynamically redistribute the accumulated energy temporally. Wang et al. addressed this condition using a PDA/PAM/GO double‐network hydrogel that combines stretchability, conductivity, anti‐freezing behavior, and self‐adhesion, as shown in Figure 7d [119]. The hydrogel was used as a piezoresistive strain sensor, the electrode of a self‐powered TENG, and the electrolyte of a flexible supercapacitor. Biomechanical motion was converted into electrical output by the hydrogel‐based TENG, rectified through a bridge circuit, and stored in the flexible supercapacitor. The stored energy powered small wearable electronic devices, including an electronic watch, an LED, and a pedometer. By storing motion‐generated energy beyond the moment of mechanical excitation, the wearable hydrogel platform links activity‐dependent harvesting to round‐the‐clock sensing availability. The hydrogel‐based system illustrates how intermittent motion energy can still be incorporated into the always‐on framework when the harvested energy is stored and redistributed by a supercapacitor. Across the cases in Figure 7, always‐on sensing is therefore less about keeping every circuit block continuously active and more about maintaining sensing availability through average‐power matching. PMIC‐based regulation and storage‐centered power supplies provide different ways to coordinate harvested power, storage capacity, sensing interval, and communication duty cycle over extended operation.

### 4.2 Event‐Driven Sensing

**FIGURE 8:**
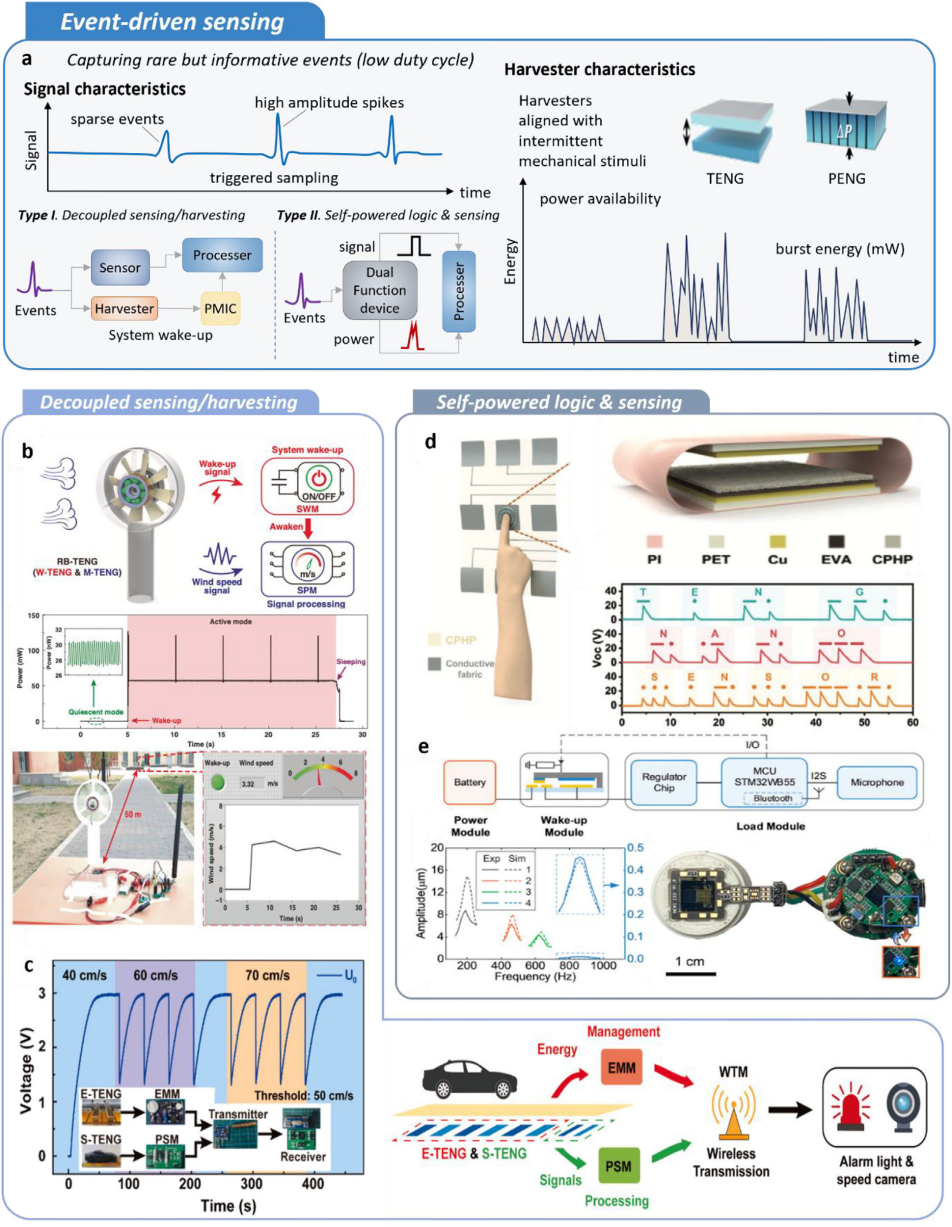
Event‐driven sensing under harvested energy. (a) Schematic description of event‐driven sensing as a low‐duty‐cycle operating paradigm for sparse but informative signals. The system remains in a low‐power watch state and activates sensing, processing, or wireless communication only when a target event is detected. Type I uses separated signal and power paths for event detection and system wake‐up, whereas Type II uses the transducer itself as both the event detector and self‐powered signal source. (b) Breeze wake‐up anemometer based on rolling‐bearing TENGs. The wake‐up TENG activates the system when the wind speed exceeds the threshold, while the measured TENG converts wind‐speed information into a frequency signal for processing and wireless transmission. Reproduced under terms of the CC‐BY license from [120]. Copyright 2024 Springer Nature. (c) Self‐powered overspeed wake‐up alarm system based on separated energy‐harvesting and overspeed‐sensing TENGs. The E‐TENG and energy management module harvest and store mechanical energy, while the S‐TENG and power‐switch module activate wireless alarm transmission only when the vehicle speed exceeds the preset threshold. Adapted with permission from [121]. Copyright 2023 Elsevier. (d) Flexible self‐powered tactile interface based on cellulose‐nanocrystal‐reinforced PVDF hybrid paper. Touch events directly generate triboelectric output signals, enabling Morse‐code‐like input, tactile trajectory recognition, and interactive control through a TENG array. Adapted with permission from [123]. Copyright 2024 Wiley‐VCH GmbH. (e) Passive self‐holding MEMS acoustic switch for zero‐power event wake‐up. Mechanical‐acoustic resonance enables frequency‐selective acoustic perception and circuit activation, while electrostatic self‐holding maintains the wake‐up state for subsequent sensing and processing. Adapted with permission from [124]. Copyright 2025 Elsevier.

A different operating paradigm is required when the target signal itself is sparse and time‐critical. Unlike continuous physiological or environmental signals that can be followed through repeated sensing windows, wind bursts, impacts, abnormal vibration, overspeed motion, acoustic signatures, and tactile inputs carry information at the moment of occurrence. As schematically shown in Figure 8a, event‐driven sensing follows a watch‐and‐burst operation. The system remains in a low‐power watch state during inactive periods and activates sensing, processing, or wireless communication only when the incoming stimulus satisfies the event condition. In this operating mode, the power path is designed to preserve system readiness with minimal standby consumption and to deliver burst energy when an event occurs. For EH‐integrated systems, matching sparse signal occurrence with short active power windows reduces the need to continuously operate the full sensing stack. Event‐driven operation can be implemented by separating the event‐detection path from the energy‐delivery path or by using the event transducer itself as the sensing and signal‐generating element. The former corresponds to Type I operation, where a lightweight trigger path wakes the system and a separate power path supports the subsequent active function. The latter corresponds to Type II operation, where the event‐generated electrical output directly serves as the sensing or control signal.

In Type I operation, the main benefit of separating the event‐detection path from the energy‐delivery path is that the system can remain dormant until the external stimulus exceeds a defined threshold. Fu et al. reported this approach using a breeze wake‐up anemometer based on rolling‐bearing TENGs, as shown in Figure 8b [120]. Two rolling‐bearing TENGs were assigned different roles. The wake‐up TENG generated the activation signal, while the measured TENG converted wind speed into a frequency signal for processing and wireless transmission. When the wind speed exceeded 2 m s^−1^, the system woke within 0.96 s and monitored wind speed. After the wind stopped, it returned to the sleep state within 0.52 s while maintaining a quiescent power below 30 nW. In this configuration, the wind event determines the timing of activation, while the separated sensing path provides the quantitative wind‐speed readout.

For alarm‐type events, the separated pathways must also support a short communication burst after threshold detection. Cao et al. implemented this operation in a self‐powered overspeed wake‐up alarm system, as shown in Figure 8c [121]. The energy‐harvesting TENG and energy management module formed the power path, while the overspeed‐sensing TENG, power‐switch module, and wireless transceiver formed the event‐triggered signal path. When the vehicle speed exceeded a preset threshold, the sensing TENG activated the power switch, and the wireless module transmitted an alarm using the harvested energy. A single wireless alarm was completed within 143 ms with an energy consumption of 0.85 mJ. The mechanical event therefore controls both the timing of system activation and the initiation of burst communication, allowing the node to remain inactive until the alarm condition is reached. The same role‐separated logic can be applied to vibration‐triggered wake‐up, where the event condition is encoded by acceleration. Lin et al. reported a self‐powered autonomous vibrational wake‐up system based on TENGs and MEMS switches [122]. The signal TENG operated as a self‐powered accelerometer, while the energy TENG supplied 0.64 mJ for wireless alarm transmission. In Type I systems, the sensing path defines whether the event condition has been reached, and the power path supplies the energy required for the subsequent active operation.

When the target event itself generates an informative electrical output, the detector and signal source can be monolithically integrated into a single transducer. Niu et al. reported this Type II route using a cellulose‐nanocrystal‐reinforced PVDF hybrid paper‐based TENG for flexible tactile perception, as shown in Figure 8d [123]. Touch events directly generated triboelectric output signals, allowing the device to operate as both an energy‐generating element and a tactile sensing unit. The pressure sensitivity of 3.95 mV Pa^−1^ and the TENG‐array demonstrations of Morse‐code‐like input, tactile trajectory recognition, and game‐character control indicate that event‐generated electrical signals can be used directly for sensing and interaction. In this route, event‐driven operation is implemented through the transducer output itself, without requiring a separate trigger path.

For event‐driven systems that monitor long inactive periods, the standby power of the monitoring path remains a key limitation. Despite the intermittent operation of primary subsystems, active sensors and signal‐processing components connected during sleep continue to drain energy via quiescent paths. Yang et al. reduced this watch‐path cost using a passive self‐holding MEMS acoustic switch for zero‐power event wake‐up, as shown in Figure 8e [124]. The switch uses mechanical‐acoustic resonance to respond selectively to target acoustic frequencies and physically connects the power module to the back‐end electronics only when the acoustic condition is satisfied. Electrostatic self‐holding maintains the wake‐up state for subsequent sensing and processing. The reported sound‐pressure threshold of approximately 1 Pa and contact resistance of 29.3 Ω indicate that acoustic event detection and electrical connection can be achieved without continuously powering the back‐end circuit.

Event‐driven operation extends signal‐power matching from average‐power delivery to event‐conditioned energy release. Low‐standby operation preserves readiness during inactive periods, while short active windows support wake‐up, sensing, processing, or wireless transmission after event detection. In practical wearable and IoT platforms, event‐triggered channels often coexist with always‐on monitoring channels and additional sensing modalities. The resulting shared‐load condition places continuous coverage and event responsiveness under the same harvested‐energy budget, motivating coordinated energy allocation across sensing, processing, and communication tasks.

### 4.3 Multi‐Modal Sensing

**FIGURE 9:**
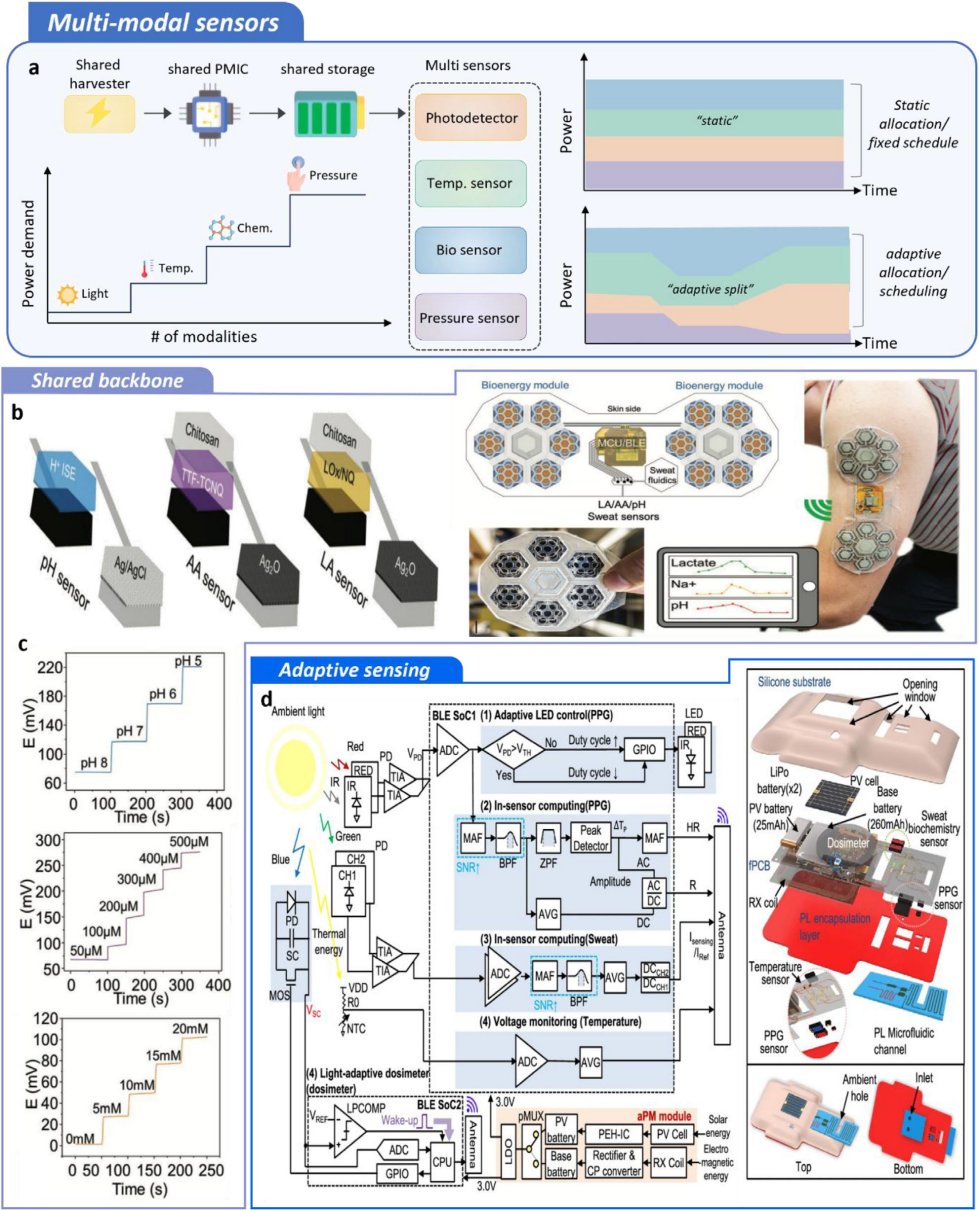
Multi‐modal sensing under harvested energy. (a) Conceptual illustration of multi‐modal sensing as a Σ_load_‐limited operation mode. Multiple sensing channels share a common harvester, PMIC, and storage element, requiring either fixed power allocation or adaptive scheduling according to the information demand and available harvested energy. (b) Wearable E‐skin microgrid with a self‐regulated bioenergy module for sweat sensing. The integrated sweat lactate biofuel cell and Zn‐AgCl battery provide a shared energy backbone for pH, ascorbic acid, and lactate sensing with wireless readout. (c) Multi‐biomarker potentiometric responses obtained from the shared‐backbone sweat sensing system. Adapted with permission from [125]. Copyright 2022 Wiley‐VCH GmbH. (d) Adaptive electronics platform for light‐based wearable sensing. Photometric sensing, PV harvesting, photoluminescent enhancement, RF charging, adaptive LED control, and in‐sensor computing are combined to reduce power consumption and wireless data transmission in multi‐modal wearable operation. Adapted under the terms of the CC‐BY license from [105]. Copyright 2025 Springer Nature.

Multi‐modal sensing emerges when sensing channels with different temporal characteristics are operated within the same EH‐integrated platform. Continuous monitoring channels require repeated sampling to preserve temporal coverage, whereas event‐triggered channels remain mostly inactive but require rapid activation when an informative signal appears. Additional sensing modalities, readout circuits, local computation, and wireless communication further reshape the total load over time. As schematically described in Figure 9a, these heterogeneous workloads are connected to a common energy path composed of the harvester, PMIC, and storage element, placing multi‐modal sensing in a Σload‐limited operating mode. Stable operation depends on how harvested energy is distributed among routine sensing, event‐triggered activation, local processing, and data transmission. This shared‐load condition motivates the use of a common energy backbone that can support multiple sensor channels within the same wearable or IoT platform.

A direct way to manage heterogeneous sensing loads is to form a shared energy backbone that provides a common power path for multiple sensor channels. In this configuration, the harvester and storage unit are designed around the voltage range, storage capacity, mechanical compliance, and readout requirements of the full sensing array. Yin et al. reported this concept using a wearable electronic skin microgrid powered by sweat lactate biofuel cells and rechargeable Zn‐AgCl batteries, as shown in Figure 9b,c [125]. The biofuel cells harvested biochemical energy from sweat, while the Zn‐AgCl batteries stored the harvested energy and supplied power to the electronics. The matched operating potentials of the two components allowed the bioenergy module to power the system without additional voltage regulation circuits. Two bioenergy modules supported wireless sweat sensing of pH, ascorbic acid, and lactate, while harvesting 2.9 J of energy overnight after 20 min of exercise and maintaining operation under repeated mechanical deformation. The shared backbone links sweat‐based energy harvesting, storage, and multi‐biomarker readout within a single mechanically compliant platform.

The backbone concept becomes more compact when the biofluid supplies both the harvested energy and the sensing sample. Ding et al. reported a fingertip‐wearable microgrid for autonomous metabolic monitoring [126]. The system integrated biofuel cells, AgCl‐Zn batteries, a flexible printed circuit board, four potentiometric electrochemical sensors, and a hydrogel‐based osmotic sweat pumping layer on a single fingertip platform. Fingertip sweat served as the biochemical fuel for energy harvesting and provided the sample for glucose, vitamin C, lactate, and levodopa sensing. The harvested bioenergy was regulated and stored in stretchable AgCl‐Zn batteries, which powered the low‐power MCU and multiplexed electrochemical readout. Full‐day operation data connected energy consumption, voltage response, and biomarker signals during daily activities, allowing energy generation and multi‐analyte sensing to be evaluated within the same operating window.

In distributed IoT systems, the shared backbone expands from biochemical readout to physical sensing channels and wireless data transfer. Wen et al. reported an all‐in‐one self‐powered wireless multi‐sensing microsystem based on a triboelectric‐electromagnetic hybrid nanogenerator [127]. The hybrid generator harvested mechanical energy from industrial‐machine vibration or human motion, stored the generated electricity through a power‐management module, and powered an MCU, signal‐processing circuit, and temperature, pressure, and UV sensors. When the storage voltage reached 3.3 V, the microsystem started automatically and transmitted sensor data through Bluetooth. The storage‐voltage waveform changed during sensor operation and wireless transmission, reflecting how sensing and communication tasks draw from the same energy reservoir. This progression from biochemical microgrids to physical IoT sensing places the shared backbone as a node‐level architecture for coordinating energy harvesting, storage, sensing, and communication.

As another approach, multi‐modal operation can be managed through adaptive sensing and allocation, where the system changes the sensing, power, computation, or communication mode according to the energy condition and signal requirement. Park et al. demonstrated an adaptive electronics platform for wireless wearable sensors that combines photometric sensing, PV energy harvesting, photoluminescent enhancement, RF power transfer, and in‐sensor computation (Figure 9d) [105]. In this platform, ambient light was used as a photometric sensing input, a PV energy source, and an optical resource controlled through photoluminescent enhancement and adaptive LED operation. The electronics selected among PV harvesting, PV battery, base battery, and RF power transfer according to the power condition, while the sensing circuit adjusted LED operation according to ambient illumination. The same platform also moved part of the computation to the BLE system‐on‐chip, extracting heart rate and blood oxygen saturation before wireless transmission. By transmitting processed parameters rather than raw photodetector signals, the data rate was reduced from 400 to 4.00 B s^−1^. In multi‐modal sensing, reducing the communication and computation load can be as important as increasing harvested power, because both directly affect the shared energy budget.

Related wearable IoMT systems further demonstrate that adaptive operation effectively mitigates the overall workload of multi‐sensor platforms. Chong et al. experimentally validated a hybrid PV and RF energy‐harvesting wearable system for measuring body temperature, heartbeat, SpO_2_, and acceleration [128]. The system used PV and RF EH to improve power availability across indoor and outdoor conditions and adopted load switching with an event‐management algorithm. With this algorithm, the current consumption decreased from 31 mA to 18.6 mA, while the maximum PV conversion efficiency reached 14.35%. The same work also described a power management unit with a broad input voltage range and task‐level software optimization for wearable eMeD devices. In this case, adaptation is applied not only to the energy source, but also to the sensing load itself, allowing the wearable node to reduce unnecessary operation when the available energy or event condition changes. At a broader IoT scale, Liri et al. developed PERMIT, a lightweight model‐predictive energy‐management framework for renewable‐energy‐powered multi‐sensor devices [129]. Their PERMIT framework decomposes the energy‐management problem into time‐dependent energy allocation and task‐dependent sensor scheduling and was evaluated using a solar‐powered Raspberry Pi equipped with camera, temperature, humidity, and soil‐moisture sensors. This algorithmic layer extends multi‐modal signal‐power matching from device architecture to task scheduling, where the sampling rate, sensing modality, and information utility are adjusted according to battery state and predicted energy input.

These adaptive strategies expand multi‐modal EH‐integrated sensing beyond the physical combination of multiple sensors. As multiple sensing channels are integrated into a single wearable or IoT platform, the limiting factor shifts toward the cumulative workload of sensing, computation, and wireless communication. Stable operation depends on how channel activation, sampling frequency, local processing, and data transmission are coordinated with the available harvested energy. This shared‐load perspective leads naturally to the broader challenges of EH‐integrated sensing systems, including power delivery, energy intermittency, event responsiveness, and energy‐aware scheduling. Table 4 summarizes the representative systems discussed in Section 4 according to their operating paradigm, power‐management strategy, dominant signal–power matching consideration, and key operation metrics.

**TABLE 4:** Representative EH‐integrated sensing systems classified by operating paradigm and signal‐power matching.

Operating paradigm	Energy source	Sensing target / modality	Power‐management / storage strategy	Dominant signal‐power matching consideration	Key operation metric	Refs.
Always‐on	Body heat / TEG	Continuous glucose monitoring	Voltage step‐up converter + Li‐S battery	Average harvested power matched to continuous sensing load	378 µW max; 83 µW supplied vs 64 µW sensor load	[117]
Always‐on	Body heat / TEG	PPG	Boost converter + 10.8 mF capacitor bank	Low‐level thermal energy accumulated for repeated sensing and BLE windows	100 Hz PPG for 3 s followed by BLE transmission	[118]
Always‐on	Body motion / TENG	Strain sensing / wearable electronics	Rectification + flexible supercapacitor	Temporal redistribution of intermittent mechanical energy	Stored energy powered watch, LED, and pedometer	[119]
Event‐driven	Wind / TENG	Wind‐speed event	Separate wake‐up TENG and measurement TENG	Low‐standby monitoring with threshold‐triggered activation	<30 nW; 0.96 s wake‐up; 0.52 s return to sleep	[120]
Event‐driven	Mechanical motion / TENG	Overspeed event	EH path + power switch + wireless transceiver	Burst‐energy delivery following threshold detection	0.85 mJ per alarm; 143 ms transmission	[121]
Event‐driven	Vibration / TENG	Acceleration / vibration event	Signal TENG + energy TENG + MEMS switch	Separated event‐detection and burst‐energy pathways	0.64 mJ supplied for wireless alarm	[122]
Event‐driven	Touch / TENG	Tactile input	Direct event‐generated triboelectric output	Event‐generated electrical output serves directly as sensing signal	3.95 mV Pa^−1^	[123]
Event‐driven	Acoustic event	Acoustic wake‐up	Passive MEMS switch + electrostatic self‐holding	Near‐zero‐power watch path with selective activation	≈1 Pa threshold; 29.3 Ω contact resistance	[124]
Multi‐modal	Sweat lactate / biofuel cell	pH, ascorbic acid, lactate	Biofuel cell + rechargeable Zn‐AgCl battery	Shared energy backbone across multiple sensing channels	2.9 J harvested overnight after 20 min exercise	[125]
Multi‐modal	Sweat / biofuel cell	Glucose, vitamin C, lactate, levodopa	Biofuel cell + AgCl‐Zn battery	Shared storage and multiplexed readout under a common energy budget	Full‐day metabolic monitoring	[126]
Multi‐modal	Mechanical vibration / hybrid nanogenerator	Temperature, pressure, UV	Power‐management module + storage + MCU	Shared reservoir for sensing, processing, and wireless transmission	Automatic system activation at 3.3 V	[127]
Multi‐modal	Light + RF	Photometric and physiological sensing	PV, batteries, RF transfer, adaptive control	Adaptive allocation among harvesting, sensing, computation, and communication	Wireless data rate reduced from 400 to 4.00 B s^−1^	[105]
Multi‐modal	PV + RF	Temperature, heartbeat, SpO_2_, acceleration	Hybrid EH + load switching	Sensing‐load adaptation according to available energy	Current reduced from 31 to 18.6 mA; PV efficiency 14.35%	[128]
Multi‐modal	Solar	Camera, temperature, humidity, soil moisture	Predictive energy allocation + task scheduling	Time‐dependent energy allocation and task‐dependent sensor scheduling	Sampling/task schedule adjusted to battery state and predicted energy input	[129]

*Note: Reported metrics are selected to reflect the system‐level operation discussed in the main text rather than to provide direct performance rankings across different device types*.

## 5 Conclusion and Outlook

Energy‐harvesting‐integrated sensors are evolving from simple self‐powered devices into coordinated sensing systems in which energy generation, storage, signal acquisition, processing, and wireless communication are operated within a shared platform. This review discussed recent progress from two connected perspectives. The first is integration architecture, where co‐packaged, monolithically integrated, and co‐functional designs determine how harvesting and sensing components are physically arranged and functionally coupled. The second is operating mode, where always‐on, event‐driven, and multi‐modal sensing require different forms of signal‐power matching. Always‐on systems rely on average‐power matching, event‐driven systems rely on low‐standby watch states and burst‐mode activation, and multi‐modal systems rely on shared‐load coordination across multiple sensing channels.

**FIGURE 10:**
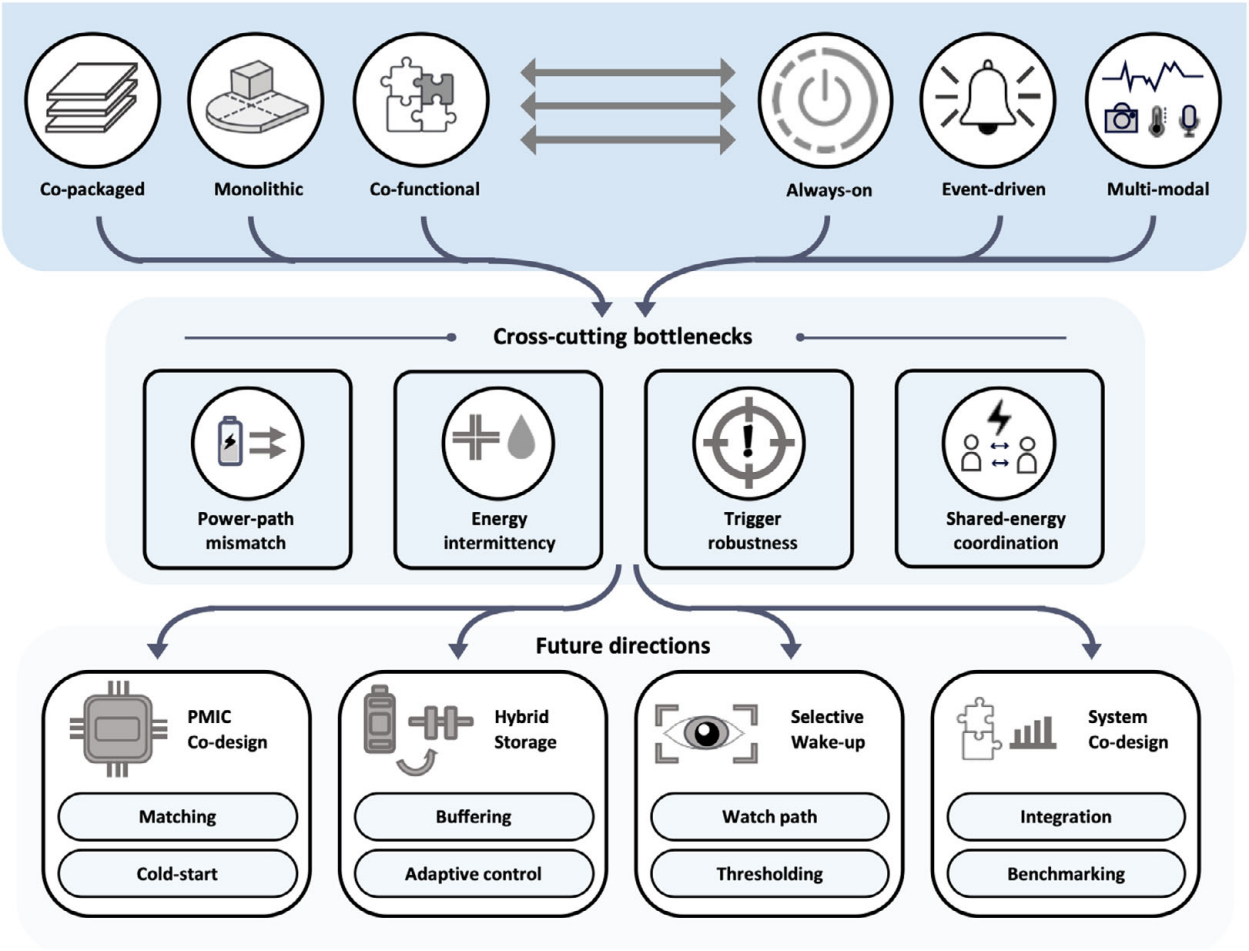
Future directions for energy harvester‐integrated sensing systems. Schematic illustration of the three integration architectures (co‐packaged, monolithic, and co‐functional) and their corresponding operating paradigms (always‐on, event‐driven, and multi‐modal) for energy harvester‐integrated sensing systems. The interaction between these structural configurations and operational modes gives rise to four cross‐cutting bottlenecks: power‐path mismatch, energy intermittency, trigger robustness, and shared‐energy coordination. To overcome these fundamental challenges and realize fully autonomous platforms, a system‐level co‐design strategy is required, emphasizing four key future directions: active PMIC co‐design, adaptive hybrid storage, selective wake‐up mechanisms, and system co‐design for comprehensive integration and benchmarking.

Despite rapid progress, several bottlenecks remain before EH‐integrated sensing systems can operate reliably in practical wearable and IoT environments, as summarized in Figure 10. A persistent challenge is the mismatch between harvester output and sensor operating requirements. Ambient harvesters often generate low, fluctuating, or impedance‐dependent outputs, while sensing, readout, and communication blocks require regulated voltage, sufficient current, and stable timing. This mismatch becomes more complex when multiple modules, interfaces, and functional layers are integrated within a compact platform. A second challenge arises from the temporal mismatch between energy generation and sensing demand. Body heat and indoor light may provide weak but relatively continuous inputs, whereas motion, vibration, airflow, and biochemical activity often appear intermittently. Stored energy therefore has to support repeated sensing windows, maintain readiness during inactive periods, and supply short bursts for wireless communication. This requirement becomes more demanding in event‐driven and multi‐modal systems, where rare events and multiple sensing loads can compete for the same energy reservoir.

Reliable event recognition is another critical issue. Event‐driven systems should remain inactive during irrelevant perturbations but respond immediately to meaningful wind bursts, impacts, abnormal vibration, overspeed motion, acoustic signatures, or tactile inputs. False wake‐up wastes stored energy, while missed events reduce sensing reliability. Selective wake‐up elements, passive switches, self‐powered threshold detectors, and mechanically or acoustically resonant devices will therefore be important for reducing standby loss while preserving event responsiveness. As more sensing channels are integrated, the main design problem shifts toward shared‐energy coordination. Multi‐modal platforms cannot be optimized by treating each sensor independently. Channel activation, sampling frequency, local computation, and wireless transmission all contribute to the total load imposed on the harvester‐storage system. Stable operation depends on how these workloads are scheduled according to available harvested energy, storage state, signal priority, and communication demand.

Practical implementation also depends on the compatibility of the fabrication processes used to integrate heterogeneous functional units. Forming energy‐harvesting, sensing, storage, and interconnection layers within a shared platform can introduce process constraints because the deposition or thermal treatment required for one component may affect adjacent materials or interfaces [92, 130]. These constraints become more relevant as the degree of integration increases and individual fabrication steps become less independent. Process compatibility, thermal budget, and fabrication yield therefore need to be considered when translating integrated device architectures toward larger‐scale manufacturing.

After deployment, variations in both the available energy source and sensing environment introduce a different set of constraints. Changes in illumination, temperature, humidity, body motion, and mechanical deformation can alter the harvested‐energy profile as well as the baseline response of the sensing unit [125]. Wearable systems can additionally experience user‐dependent differences in physiological and motion‐derived inputs, while distributed IoT nodes need to coordinate sensing and wireless communication under variable energy availability [128]. These operating variations change the balance among harvesting, storage, sensing, and communication, increasing the need for adaptive power allocation under realistic deployment conditions.

Future progress will require co‐design across circuits, devices, and systems. Active PMIC‐harvester co‐design can stabilize weak EH outputs by matching impedance, start‐up voltage, conversion efficiency, and duty‐cycle control to the specific harvester and sensing workload. Adaptive hybrid storage systems can combine batteries, supercapacitors, stretchable capacitors, or material‐integrated storage units to support both average‐power operation and burst‐mode activation. Selective wake‐up mechanisms can reduce the cost of long standby periods, while system‐level scheduling can distribute energy across always‐on, event‐triggered, and multi‐modal sensing tasks. The evaluation of EH‐integrated sensors should also move beyond isolated metrics such as peak output power, responsivity, or sensitivity. More relevant benchmarks include sensing availability, wake‐up reliability, energy per event, recovery time, communication duty cycle, storage retention, and operation under realistic ambient‐energy profiles. These metrics better reflect whether harvested energy is matched to the temporal structure of sensing, computation, and communication.

The broader opportunity of EH‐integrated sensing lies in treating energy harvesting and sensing as a single coupled design issue. By connecting integration architecture with operating mode and energy‐management strategy, self‐sustaining sensing platforms can move toward long‐term wearable healthcare, distributed environmental monitoring, industrial diagnostics, and autonomous IoT networks with reduced dependence on conventional battery replacement.

## Author Contributions

**Taehyun Park**: investigation, funding acquisition, Writing – original draft, Writing – review and editing, visualization, validation, conceptualization. **Ki‐Yoon Park**: investigation, writing – original draft, writing – review and editing, visualization, validation, conceptualization. **Yeonho Choi**: investigation, writing – original draft, writing – review and editing, validation, visualization, conceptualization. **Dong‐Myeong Shin**: writing – original draft, writing – review and editing, validation. **Hong‐Joon Yoon**: conceptualization, investigation, funding acquisition, writing – original draft, writing – review and editing, visualization, validation, project administration, supervision, resources. **Hocheon Yoo**: conceptualization, investigation, funding acquisition, writing – original draft, writing – review and editing, visualization, validation, project administration, resources, supervision.

## Conflicts of Interest

The authors declare no conflicts of interest.
